# Motivational Interdependence in Couple Relationships

**DOI:** 10.3389/fpsyg.2022.827746

**Published:** 2022-05-23

**Authors:** Sebastian Pusch, Felix D. Schönbrodt, Caroline Zygar-Hoffmann, Birk Hagemeyer

**Affiliations:** ^1^Institut für Psychologie, Friedrich-Schiller-Universität, Jena, Germany; ^2^Department Psychologie, Ludwig-Maximilians-Universität München, Munich, Germany

**Keywords:** interdependence, motives, couple relationship, interpersonal perception, communion, response surface analysis

## Abstract

This article presents an integrative conceptual model of motivational interdependence in couples, the MIC model. Based on theoretical tenets in motivation psychology, personality psychology, and research on interpersonal perception, the MIC model postulates that two partners' motive dispositions fundamentally interact in shaping their individual motivation and behavior. On a functional level, a partner's motivated behavior is conceptualized as an environmental cue that can contribute to an actor's motive expression and satisfaction. However, the partner's motivated behavior is considered to gain this motivational relevance only via the actor's subjective perception. Multilevel analyses of an extensive experience sampling study on partner-related communal motivation (*N* = up to 60,803 surveys from 508 individuals nested in 258 couples) supported the MIC model. Participants, particularly those with strong communal motive dispositions, behaved more communally at moments when they perceived their partners to behave more communally. In addition, participants experienced momentary boosts in satisfaction when they behaved more communally and, at the same time, perceived their partners' behavior as similarly communal. Broader implications of the MIC model for research on romantic relationships are discussed.

## 1. Introduction

Partner-related motives describe dispositional preferences for specific classes of end states, or incentives, that are tied to one's romantic relationship and partner^1^ (such as intimacy). As individual-difference variables, they describe the extent to which people typically experience certain relationship situations as fulfilling and meaningful. Dual-motive theory (McClelland, 1987; Schultheiss, 2001) suggests that partner-related motives are represented in two functionally distinct motive systems. Explicit motives, on the one hand, reflect a person's motivational self-concept. Due to their propositional representation, explicit motives can be verbalized as personal goals and desires whose realization provides eudaimonic rewards such as self-validation and inner coherence (Cantor and Malley, 1991). Implicit motives, on the other hand, are rooted in hedonic rewards (Brunstein et al., 1998; Hofer and Busch, 2011) and operate largely outside conscious awareness. Implicit motives reflect learned associative networks linking situational cues and instrumental behaviors with affectively charged incentives. Explicit and implicit motives are important predictors of relationship functioning. They are associated with how people interact with their partners (Zygar et al., 2018; Zygar-Hoffmann et al., 2020b), how they perceive their partners (Pusch et al., 2020, 2021), how satisfied they are with their relationships (Hagemeyer and Neyer, 2012; Hagemeyer et al., 2013b; Zygar et al., 2018), and how long their relationships last (Hagemeyer et al., 2013a).

However, looking only at the experiences and behavior of one partner paints too narrow a picture of motivation in couple relationships. Romantic partners' individual motivational processes are increasingly recognized as fundamentally interdependent. Previous studies suggest that partners' individual motives contribute not only to their own but also to each other's relational outcomes (e.g., Sanderson and Cantor, 2001; Hagemeyer et al., 2013a; Zygar et al., 2018). Couple members seem to report highest satisfaction and wellbeing if their individual motives match (Meyer and Pepper, 1977; Le and Agnew, 2001; Feeney, 2004; Riediger and Rauers, 2010; Arránz Becker, 2013; Czikmantori et al., 2018), whereas frustration and discord may arise if their motives interfere with each other (Drigotas and Rusbult, 1992; Gere and Schimmack, 2011; Righetti et al., 2016; Gere and Impett, 2017).

Although existing research has provided important insights into motivational interdependence in couples, little is known about the functional processes underlying everyday motive transactions between partners. To fill this gap, the current article presents an integrative model of Motivational Interdependence in Couples, the MIC model. Based on general assumptions of motivation psychology, personality psychology, and research on interpersonal perception, the MIC model explicates how two partners' motive dispositions can—by virtue of their perceptual and behavioral functions—jointly shape individual behavior and need satisfaction. After introducing the MIC model, we present an extensive experience sampling study applying the model to the domain of partner-related communion motivation.

## 2. The Motivational Interdependence in Couples (MIC) Model

**Figure 1:**
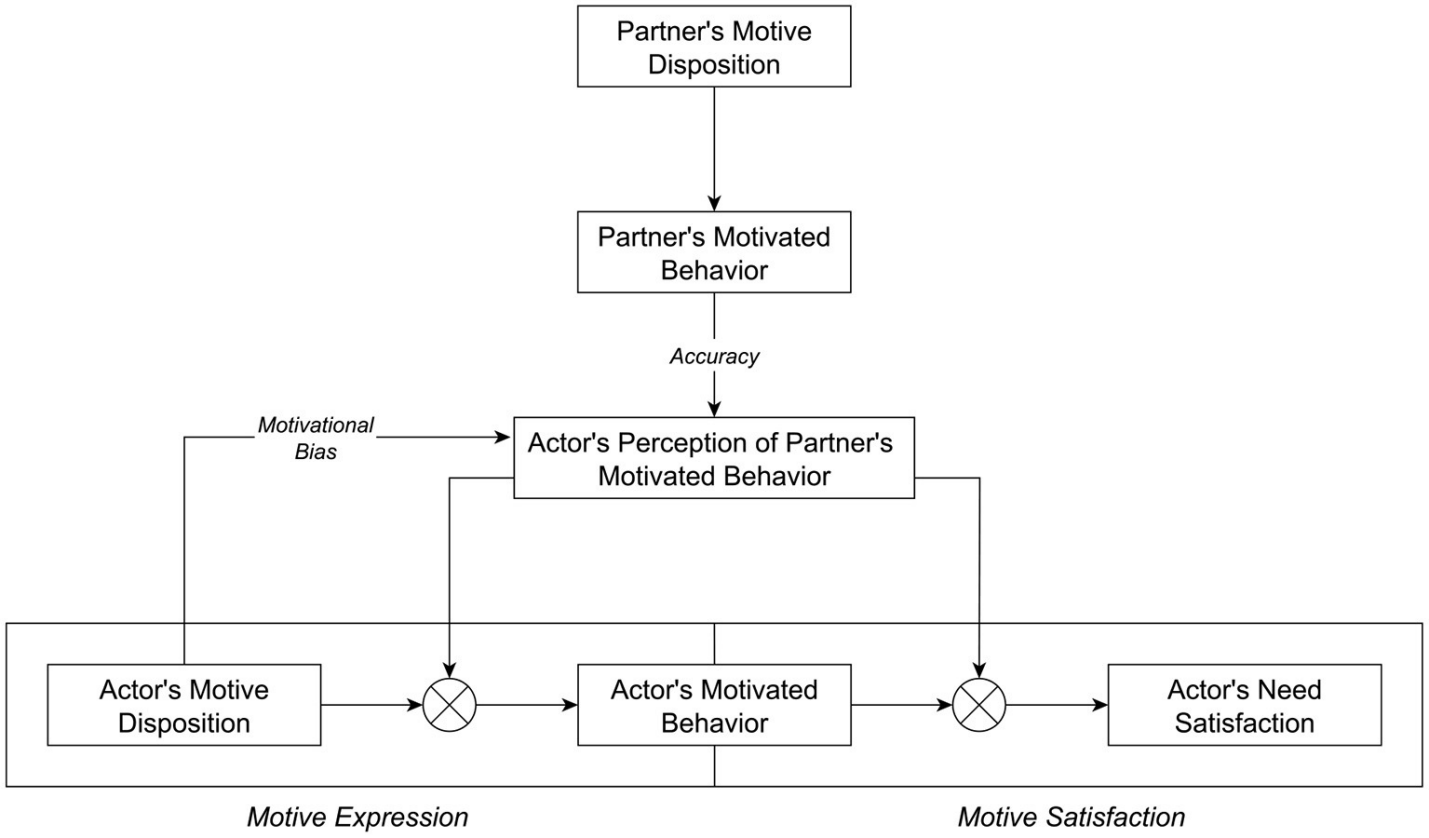
The motivational interdependence in couples model. Figure available at: https://osf.io/2fz5w/ under a CC-BY4.0 license.

The MIC model focuses on the functional underpinnings of interdependence between the motive dispositions of romantic partners. The model is illustrated in Figure 1, depicting the motivational process of an actor and how their partner can contribute to this process. In a nutshell, the MIC model conceptualizes the partner's motivated behavior as a situational cue that can become effective at two distinct phases of the actor's motivational process. First, during the *Motive Expression* phase, the partner's motivated behavior can arouse the actor's motive and thereby contribute to the instigation of the actor's motivated behavior. During the second *Motive Satisfaction* phase, the partner's motivated behavior can present either opportunities or obstacles for the actor's motivated behavior and thereby promote or hinder need satisfaction, respectively. However, the partner's motivated behavior can only gain this motivational relevance via the actor's subjective *perception of the partner's motivated behavior*. Although this partner perception can represent an accurate representation of the partner's actual behavior, it can also be biased by the actor's own motive disposition. Hence, interdependence between two partners' motives is attributed to their behavioral and perceptual functions in a dyadic context. The MIC model takes the perspective of an actor whose motivation is affected by cues provided by their partner. We maintain the actor-partner distinction throughout this paper. However, actors and partners are meant to represent the two interchangeable members of a couple, as both members can assume both roles. Therefore, the model must be understood as dyadic and recursive at heart. Moreover, the MIC model does not negate the occurrence of main effects (e.g., engaging in motivated behavior can be intrinsically rewarding in some cases). Rather, it centers on those motivational processes that interlink actors' and their partners' individual behaviors and experiences in the relationship.

The MIC model builds on four theoretical assumptions, which are shown in Table 1. These assumptions originate from different fields of psychological research and refer to rather general tenets about motivation, personality, and interpersonal perception. The following sections provide an outline of the respective assumptions before turning to their implications for couples' motivational interdependence. Each section presents examples and findings from the domain of partner-related communion motivation, which was the focus of our empirical investigation. Partner-related communal motives are defined as relatively stable evaluative dispositions for classes of relationship situations that share communal characteristics such as experiences of emotional and physical closeness to the partner, companionship, or feeling as part of a dyad (Hagemeyer and Neyer, 2012). Previous studies have shown that communal motives are positively associated with relationship quality (Hagemeyer et al., 2013a,b; Czikmantori et al., 2018; Zygar et al., 2018), relationship stability (Hagemeyer et al., 2013b), communal partner behavior (Zygar et al., 2018; Zygar-Hoffmann et al., 2020b), and positively biased partner perception (Pusch et al., 2020, 2021). However, the MIC model can be applied to other partner-related motives as well. In the final section of this introduction, we outline how.

**Table 1:** Theoretical assumptions and their implications for motivational interdependence in couple relationships.

**Assumptions**			**Implications**
1.	Person × Situation	Motivation is the product of the interplay between a person's motive dispositions and situational stimuli.	The partner provides important situational stimuli through observable motivated behavior. Thus, the actor's motivation is a product of the interplay between the actor's motive dispositions and the partner's motivated behavior.
2.	Motivational cues	Whether situational stimuli gain relevance for a person's motivation as cues depends on their subjective incentive value.	Not the partner's actual motivated behavior is relevant for the actor's motivation, but how complementary the actor perceives it to his/her own motivation.
3.	Accuracy and bias	Interpersonal perception is both accurate and biased.	An actor's perception of the partner's motivated behavior is affected by the partner's actual motivated behavior and the actor's own motive dispositions.
4.	Motivational phases	Cues can become effective in two distinct phases of the person's motivational process:	Partner perceptions can become effective in two distinct phases of the actor's motivational process:
	(a) Motive expression	Cues can arouse a person's motive dispositions and thereby contribute to the instigation of motivated behavior.	Partner perceptions can arouse the actor's motive dispositions and thereby contribute to the instigation of the actor's motivated behavior.
	(b) Motive satisfaction	Cues can present opportunities or barriers for the realization of a person's motivated behavior and thereby contribute to need satisfaction or frustration, respectively.	Partner perceptions can present opportunities or barriers for the realization of the actor's motivated behavior and thereby contribute to the actor's need satisfaction or frustration, respectively.

### 2.1. Assumption 1: Person × Situation

A central assumption in motivation psychology is that the expression and satisfaction of motive dispositions depend on situational conditions (e.g., McClelland, 1987). According to this Person × Situation approach, motivation (i.e., a person's current state of orientation toward and striving for a specific end state) and behavior are products of the interplay between a person's motive dispositions and situational stimuli tied to motive-specific incentives, so-called motivational cues. For example, previous research showed that individuals with stronger intimacy motives more often self-disclose when interacting with close others (e.g., Craig et al., 1994).

#### 2.1.1. Implications for the MIC Model

What does the Person × Situation assumption imply for couples' motivational interdependence? Partners can provide important situational stimuli through their observable behavior (Holmes, 2002; Fitzsimons and Bargh, 2003; Shah, 2003; Asendorpf, 2020) such as verbal utterances, facial expressions of emotions, or touches (Back et al., 2011), to name a few. Partner stimuli usually reflect expressions of the partner's own motive dispositions (Horowitz et al., 2006). Thus, an actor's motivation can result from the interplay between the actor's motive dispositions and the partner's motivated behavior (*Person* × *Situation* implication in Table 1). For instance, actors with strong communal motives should experience a strong motivation to spend time with their partners in situations when their partners act particularly warm and caring.

### 2.2. Assumption 2: Motivational Cues

Situational stimuli do not assume motivational relevance per se. Motivation theory posits that situational stimuli need to be subjectively perceived and linked with motive-specific incentives to function as motivationally relevant *cues* (McClelland, 1987; Schultheiss, 2001; Heckhausen and Heckhausen, 2018)^2^. The motivational relevance of situational stimuli supposedly results from learning processes starting in childhood. Through experiences of reward and punishment in the pursuit of natural incentives and acquired goals, children learn which situations and behaviors afford pleasure and pain (McClelland and Pilon, 1983; McClelland, 1987). Over time, these experiences condense into complex contingency networks linking situational stimuli with desired incentives and behaviors instrumental to their attainment. Differentiation of motives *via* ongoing learning processes can also occur later in life, especially concerning individual implementation styles, for instance, regarding the kind of relationship in which a person's motives are preferably expressed. Because they are linked to motive-specific incentives in this way, situational cues affect a person's motivation. Hence, whether situational stimuli gain relevance for a person's motivation as cues, depends on how the person subjectively perceives them.

#### 2.2.1. Implications for the MIC Model

The MIC model states that interpersonal perception assumes a crucial role in couples' motivational interdependence. Perception determines which behaviors of the partner gain motivational relevance for the actor's motivation. Thus, not the partner's actual motivated behavior, but how the actor subjectively perceives it, provides motivational cues for the actor (*Motivational Cues* implication in Table 1).

In the actor's eye, partner behavior must hold the potential for need satisfaction to function as a motivational cue. Interpersonal theory states that needs are satisfied if actors' and their partners' behaviors combine in a complementary manner (Winch et al., 1954; Leary, 1957; Carson, 1969). According to Horowitz et al. (2006), an actor's motive disposition can energize interpersonal behavior, which in turn “invites” the partner's complementary reaction to satisfy the actor's motive disposition. In the communion domain, complementarity is established by similarity: To attain communion, actors need to see their partners as similarly interested in communion. For example, studies have found perceived partner responsiveness to be an important predictor of communal intimacy (Reis et al., 2004). Partner responsiveness describes a partner's caring, understanding, and validating reaction toward the actor's communication of needs and wishes. From a motivational standpoint, perceived partner responsiveness may act as a cue that can contribute to the actor's communal motivational processes. Similarly, previous research has shown that how people perceive and interpret their partner's support is more relevant for relationship functioning than objective assessments of partner support (Brunstein et al., 1996; Uchino et al., 1996). Also, Murray et al. (2002) showed that couple members are happier in their relationships the more similar they perceive their own and their partners' interpersonal qualities. Actual similarity did not contribute to satisfaction beyond perceived similarity, a finding that has also been reported for the perception of broader personality traits such as the Big Five (Furler et al., 2014), values (Murray et al., 2002), or emotions (Sels et al., 2020).

To summarize, the MIC model postulates that actors need to subjectively perceive their partners' behavior as complementary for it to gain motivational relevance as a cue. Partner behavior is complementary when it holds—in the actor's eyes—the potential to promote the actor's incentive attainment.

### 2.3. Assumption 3: Accuracy and Bias

The way we perceive others can be both accurate and biased. In romantic relationships, there is strong evidence for both (Gagné and Lydon, 2004; Fletcher and Kerr, 2010). On the one hand, there seems to be substantial agreement between actors' perceptions of their partners' characteristics and partners' self-ratings, which are commonly used as an accuracy criterion (Fletcher and Kerr, 2010). On the other hand, people also tend to see their partners in an overly positive light (Murray et al., 1996) and project their ideal conceptions of a partner or their own attributes onto their partners (Kenny and Acitelli, 2001; Lemay et al., 2007; Lemay and Clark, 2008). Importantly, bias and accuracy must not be seen as mutually exclusive but rather as coexisting phenomena that take on distinct functions in the relationship (Gagné and Lydon, 2004; Luo and Snider, 2009; Fletcher and Kerr, 2010).

#### 2.3.1. Implications for the MIC Model

Actors' perceptions of their partners' motivated behavior should be rooted in both reality and wishful thinking. On the one hand, actors' should hold a partly accurate view of their partners' actual motivated behavior. On the other hand, partner perceptions should also be susceptible to perceptual biases, which can originate from the actors' own motive dispositions (*Interpersonal Perception* implication in Table 1).

A meta-analysis by Fletcher and Kerr (2010) found evidence that people are able to accurately perceive their partners' behavior, but also show substantial biases in their perception. Similarly, Pusch et al. (2020) showed that people can judge their partners' momentary communal behavior with considerable accuracy. However, independent of this accuracy, people with stronger communal motives ascribed more communion to their partners' behavior than their partners' did themselves. Converging evidence for accuracy and motivational bias was also found in research on partner perceptions of daily goals (LaBuda et al., 2019) and dispositional motives (Sanderson and Cantor, 2001; Pusch et al., 2021). These findings corroborate that actors' perceptions of their partners' motivational strivings are both accurate and biased by the actors' own motives.

### 2.4. Assumption 4: Motivational Phases

How exactly do motivational cues affect a person's motivational process? Most theorists agree that motivational processes involve at least two phases (Schultheiss and Wirth, 2018): A *motive expression* phase, during which behavior is energized to attain a motive-specific incentive, and a *motive satisfaction* phase, during which the rewards from attaining an incentive are reaped. Situational cues can affect both motive expression and satisfaction.

As detailed above (Section Assumption 2: Motivational Cues), cues refer to stimuli that a person subjectively links with motive-specific incentives and instrumental behavior to attain these incentives. Such behavior-reward contingencies play a central role in a person's motive expression: When situational cues trigger the anticipation of a motive-specific incentive, motivated behavior aimed at attaining this incentive is energized (McClelland, 1987; Schultheiss, 2001). Hence, one way in which situational cues can affect a person's motivation is by contributing to the expression of motive dispositions into behavior. However, merely engaging in motivated behavior does not guarantee success in attaining the desired incentive required for motive satisfaction. Intermediate situational influences—including the perceived behavior of others—can pose either opportunities or barriers for the person's motivated behavior. Hence, a second way for situational cues to affect a person's motivational process is to foster or hinder the realization of a motive-specific incentive.

#### 2.4.1. Implications for the MIC Model

The two phases of motive expression and motive satisfaction offer valuable insights into the functional underpinnings of couples' motivational interdependence. We propose that both phases can be affected by perceived motivated partner behavior (*Motivational Phases* implication in Table 1).

Sometimes, a single perceived partner behavior can contribute to both motive expression and satisfaction. For instance, perceiving the partner to be genuinely interested in one's feelings and thoughts during a conversation should not only drive actors with strong communal motives to show more communal behavior (e.g., self-disclosure), but also contribute to actors' communal need satisfaction (e.g., feeling close and cared for). In other contexts, perceived partner behavior affects only one motivational phase. For instance, perceiving care and support by the partner can satisfy the actor's communal needs, but the actor may seek closeness in the first place due to relationship-external stressors (e.g., stress at work). Conversely, a perceived partner behavior that contributes to motive expression may sometimes have no relevance for motive satisfaction. For example, although the partner's suggestion to spend time together may motivate the actor to meet with the partner, this mere suggestion should hardly satisfy the actor's communal needs. What should matter more for communal motive satisfaction is the partner's perceived behavior during the shared time (e.g., whether the partner appears to genuinely enjoy the time together).

##### 2.4.1.1. Motive Expression

The MIC model proposes that during the motive expression phase, partner perceptions can contribute to the energization of motivated behavior (*Motive Expression* implication in Table 1). For example, consider a situation where the partner asks the actor to meet and spend time together. If the actor has a strong communal motive and perceives this question to indicate the attainability of companionship with the partner (a communal incentive), the actor will most likely be motivated to meet the partner.

Partner cues seem to motivate complementary behavioral reactions (Horowitz et al., 2006). For example, many studies indicate that communal partner behavior invites similarly communal behavior (Sadler and Woody, 2003; Markey and Markey, 2007; Sadler et al., 2009; Markey et al., 2010). The MIC model proposes that such invitations correspond to the mechanism of motive expression: If an actor links the partner's behavior to communal incentives, this perception can arouse the actor's communal motives and thereby instigate the actor's communally motivated behavior.

##### 2.4.1.2. Motive Satisfaction

During the motive satisfaction phase, partner perceptions can present either opportunities or barriers to need satisfaction (*Motive Satisfaction* implication in Table 1). Communal experiences can only be realized if the actor perceives both couple members to act in concert (Laurenceau et al., 2005)—the actor's expressions of affection need to be embraced and reciprocated by the partner. If the partner does not reciprocate the actor's communal behavior, the actor's communal motives may be frustrated.

In a recent experience sampling study, Zygar et al. (2018) asked participants multiple times per day to report their current communal motivation, current relationship satisfaction, and recent activities with their partners. Individuals who reported high communal motivation at one assessment point were more satisfied with their relationships at the subsequent assessment the more communal they rated their activities with their partners in the meantime. In contrast, the lack of communal exchanges led to momentary dips in relationship satisfaction. Indeed, perceiving the partner to react in a complementary manner toward one's communal striving appears to be a crucial source of relationship quality (Markey and Markey, 2007). Moreover, sensing that one's partner is supportive and understanding of one's needs appears to be beneficial not only for partner-related need satisfaction but also for personal wellbeing (Brunstein et al., 1996; Uchino et al., 1996; Feeney, 2004; Salmela-Aro et al., 2010; Fitzsimons and Finkel, 2011).

### 2.5. Summary

Previous research corroborates that couple members' motive dispositions jointly shape their individual behavior and experiences in the relationship (Meyer and Pepper, 1977; Riediger and Rauers, 2010; Gere et al., 2011; Arránz Becker, 2013; Righetti et al., 2016; Gere and Impett, 2017; Czikmantori et al., 2018; Denzinger et al., 2018). Integrating theoretical tenets and empirical findings from motivation psychology, personality psychology, and interpersonal perception research, the MIC model provides a functional explanation for this interdependence. Through observable motivated behavior, the partner is proposed to provide important situational cues for the actor's motive expression and satisfaction. Partner cues should therefore contribute to the actor's motivated behavior and need satisfaction, respectively. However, the partner's motivated behavior is supposed to gain this motivational relevance only if the actor perceives it as complementary to their own motivational strivings.

This functional approach enables the formulation of testable hypotheses about how people enact their motive dispositions in interaction with their partners. The model can be used to predict under what conditions (i) people engage in a certain motivated partner-related behavior and (ii) feel satisfied with their partners and relationships: Both depend on whether people perceive their partners' behavior as complementary to their own motivational strivings.

## 3. The Present Research

The present research examined the empirical usefulness of the MIC model for the domain of partner-related communal motives. We focused on communion motivation for several reasons. Communal experiences such as closeness and intimacy with the partner appear to be a cornerstone of happy and long-lasting relationships and an important source of personal health and wellbeing (Hassebrauck and Fehr, 2002; Reis and Aron, 2008; Holt-Lunstad et al., 2010; Frost and Forrester, 2013). In addition, there is strong evidence of the interdependent nature of communion in romantic relationships in that it can only be realized if both couple members seek it (Reis and Shaver, 1988; Surra and Longstreth, 1990; Laurenceau et al., 2005). An increasing number of studies corroborates that partner-related communal motives are associated not only with individuals' own but also their partner's relational wellbeing (Sanderson and Cantor, 2001; Hagemeyer et al., 2013a; Czikmantori et al., 2018). Moreover, people seem to be particularly prone to reciprocate the communal behavior of their partners, which in turn was found to foster relationship quality (Markey and Markey, 2007; Markey et al., 2010). The MIC model posits that people can express and satisfy their motives to the extent that they perceive their partners' motivational strivings as complementary to their own. In the communion domain, complementarity is expressed in similarity (Carson, 1969; Horowitz et al., 2006)—strong communal motives are best satisfied if the partners' communal motivation is perceived as similarly strong (Reis and Shaver, 1988; Laurenceau et al., 2005). Thus, the more people perceive their partners as similarly communion-oriented, the better conditions they should find to express and satisfy their own communal motives.

Real-life partner behavior most often reflects a mix of both verbal and nonverbal behavior (e.g., verbal support for the partner likely comes along with nonverbal expressions of sympathy). Hence, communal motivational interdependence between couples may occur on both an explicit and an implicit level, likely simultaneously. Whereas, explicit motives should regulate the verbalizable and cognitive aspects of motivational interdependence more (e.g., partners' shared deliberate decisions on living arrangements; Hagemeyer et al., 2015), implicit motives should mainly regulate the less verbalizable, affective aspects of motivational interdependence between partners (e.g., shared emotional experiences; Dufner et al., 2015). In our analyses, we therefore considered both participants' explicit and implicit communal motive dispositions. As indicators of explicit and implicit communal motives, we assessed participants' explicit partner-related desires for closeness (Hagemeyer et al., 2013b) and implicit partner-related needs for communion (*pnCommunion*; Hagemeyer and Neyer, 2012), respectively.

### 3.1. Hypotheses

We applied the MIC model to data from an extensive experience sampling study asking participants to report their momentary behavior, thoughts, and feelings five times per day for 4 weeks. This enabled us to capture communal motivational interdependence as it occurs in couples' everyday lives. We focused our analyses on the two central interdependence mechanisms in the MIC model: Motive expression and motive satisfaction. Our hypotheses were derived from the corresponding theoretical assumptions and their implications for couples' motivational interdependence presented in Table 1. First and consistent with the *Motive Expression* implication, we expected that actors' momentary perceptions of their partners' communal behavior can arouse actors' communal motive dispositions and thereby contribute to actors' own communal behavior. That is, perceived communal partner behavior should elicit similar communal behavior of the actor (Horowitz et al., 2006), and this association should be even stronger for actors with stronger communal motive dispositions.

*H1: Actors' momentary communal behavior is positively predicted by the interaction between their communal motive dispositions and their perceptions of their partners' momentary communal behavior*.

Second and consistent with the *Motive Satisfaction* implication, we assumed that perceived communal partner behavior can, in turn, contribute to actors' momentary communal need satisfaction. Research suggests that communal needs are best satisfied when interaction partners behave in a similar communal manner (e.g., Carson, 1969; Horowitz et al., 2006; Markey and Markey, 2007; Sadler et al., 2009). Accordingly, the MIC model proposes that perceived similarity in communal behavior should provide most benefits for need satisfaction (*Motivational Cues* implication). We therefore hypothesized that individuals would experience boosts in communal need satisfaction at moments when they behave more communally and, at the same time, perceive their partners' behavior as similarly high in communion.

*H2: Actors' momentary communal need satisfaction is positively associated with their own momentary communal behavior, their perceptions of their partners' momentary communal behavior, and similarity between the two*.

Communal need satisfaction usually generates feelings of pleasure and fulfillment that can rub off on overall satisfaction with the relationship (Aron et al., 1992; Frost and Forrester, 2013). We thus additionally examined the joint effects of individuals' communal behavior and perceptions of their partners' communal behavior on momentary relationship satisfaction. Like in the prediction of need satisfaction, we assumed that participants would report higher relationship satisfaction at moments when they behave more communally and perceive their partners' behavior as similarly communal.

*H3: Actors' momentary relationship satisfaction is positively associated with their own momentary communal behavior, their perceptions of their partners' momentary communal behavior, and the similarity between the two*.

Hypotheses 2 and 3 predict that actors are more satisfied at moments when they perceive both their own and their partners' behavior to be higher in communion and more similar. Not only perceived similarity, but also the level of communal behavior should contribute to satisfaction. Traditional approaches, such as moderated regression analyses using product-interaction terms, can only model linear effects of perceived similarity on an outcome variable. Therefore, we instead applied polynomial regression and response surface analysis to test Hypotheses 2 and 3. Response surface analysis is increasingly recommended as the tool of choice for testing (perceived) similarity effects (Edwards and Parry, 1993; Schönbrodt, 2016; Barranti et al., 2017; Humberg et al., 2018; Schönbrodt and Humberg, 2018; Schönbrodt et al., 2018), as it allows to address the consequences of matches and mismatches at varying levels of the two predictors. Moreover, response surface analysis can explore additional association patterns such as an optimal discrepancy between actors' own and their partners' perceived communal behavior. Hypotheses 2 and 3 correspond to specific response surface analysis patterns which we detail in our model descriptions.

## 4. Method

The present research used data from an experience sampling study with German couples. The data are available as a scientific use-file (Zygar-Hoffmann et al., 2020a) and have been used in previous publications (Pusch et al., 2020; Zygar-Hoffmann and Schönbrodt, 2020; Zygar-Hoffmann et al., 2020b; Schönbrodt et al., 2021). However, none of these previous publications addressed the hypotheses tested in the present research, but focused on other hypotheses. We set up a permanent online repository at https://osf.io/2fz5w/, which stores reproducible R-scripts, a list of all used measures, and figures. Data handling, analyses, and plotting were carried out in R statistical environment version 4.0.2 (R Core Team, 2020) using the packages *dplyr* (Wickham et al., 2019), *stringr* (Wickham, 2019), *psych* (Revelle, 2021), *robustbase* (Maechler et al., 2021), *xtable* (Dahl, 2016), *lmerTest* (Kuznetsova et al., 2017), *lme4* (Bates et al., 2015), *RSA* (Schönbrodt and Humberg, 2018), *emmeans* (Lenth, 2021), *ggplot2* (Wickham, 2016), *RColorBrewer* (Neuwirth, 2014), and *cowplot* (Wilke, 2020)^3^. The experience sampling study is part of a larger research project on motives in couple relationships. In planning the project, we aimed for at least 80% power to detect effects of average size in psychological research (*r* = 0.21; Richard et al., 2003). On the couple level, this required at least 175 couples. The current sample largely exceeded this benchmark. Sample size was in addition determined by funding limits (e.g., monetary compensation for participants) and feasibility of the experience sampling designs (e.g., the maximum number of experience sampling surveys).

### 4.1. Participants and Procedure

German opposite-sex couples were recruited in 2017/2018 via local and online advertising. First, each member of a couple completed an online questionnaire set up via the former survey framework (Arslan et al., 2019), in which their communal motive dispositions were assessed. Next, participants were asked to download an experience sampling app (Tellmi) on their smartphones that was developed for this study. For the following 4 weeks, participants were asked to report on their momentary behavior and experiences via this app five times per day. Each day, data collection took place over a fixed period of 10–16 h, which couples scheduled beforehand. To avoid expectancy effects, invitations to the first four surveys of each day were sent at semi-random time points throughout the day (i.e., exact timing varied randomly around evenly distributed time intervals). The invitation to the evening survey was sent at a fixed time. The two partners of a couple received all survey invitations at the same time but were instructed to complete the surveys individually. The surveys were accessible for 45 min (5 h for the evening survey, because participants were instructed to finish it before going to bed). Median completion time was 2.70 min. Participants were compensated with feedback about their results and up to € 190 per couple (depending on the total number of surveys they completed). The study further included a follow-up online questionnaire to which participants were invited 1 year after completing the entry questionnaire. Data from this follow-up assessment were not used for the present analyses.

To be eligible for participation, individuals had to be involved in an opposite-sex relationship and own a smartphone that was compatible with the Tellmi-app used for ESM data collection. Participants were informed about the broad aims of the study and their consent was obtained. In total, 576 participants completed the entry questionnaire. Data exclusions were based on preregistered criteria (see https://osf.io/fhtw5/): We excluded participants who did not participate in the experience sampling or failed to complete at least one third of their experience sampling surveys (*n* = 66). One couple was excluded because both couple members' individual reports of their gender and relationship length were inconsistent across the entry and follow-up questionnaires (see https://osf.io/6v2rw/ for details). Moreover, we excluded single experience sampling surveys that were discussed with the partner (*n* = 171), collected at night-time due to a software error (*n* = 26), or answered in <1 min (*n* = 1858). The final experience sampling sample comprised 508 participants (50.2% female) from 258 couples. The mean response rate during the experience sampling was 88%, providing data from up to *N* = 60,803 observations. On average, participants were 31.44 years old (*SD* = 9.53, range = 18–68 years) and in the relationship with their current partner for 6.40 years (*SD* = 6.42, range = 2 months to 33.17 years). Roughly one third of the participants (32%) had one to four children, and 327 participants (64%) held a high-school degree (German Abitur).

### 4.2. Measures

#### 4.2.1. Entry Questionnaire: Explicit Desire for Closeness to the Partner

Participants' explicit desires for closeness to their partners were measured with the ABC questionnaire of social desires (Hagemeyer et al., 2013b). On four items each, participants rated the frequency (1 = *never* to 7 = *always*) of appetitive motivation (e.g., “I like being very close to my partner”) and aversive motivation (e.g., “I avoid being very close to my partner”; reversed) they experienced in relation to closeness with their partners. An average score across all eight items (using reversed aversion items) was computed. Internal consistency was α = 0.85 for men and α = 0.92 for women.

#### 4.2.2. Entry Questionnaire: Implicit Partner-Related Need for Communion

Participants' implicit partner-related need for communion (pnCommunion) was measured with the Partner-Related Agency and Communion Test (PACT; Hagemeyer and Neyer, 2012). This test comprises eight picture cues in the form of blurred photographs and line drawings of social scenes. Participants were instructed to invent a story about a couple in response to each picture and describe the behavior and feelings of the story's protagonist. Three questions guided participants' descriptions: “What is important to the person in this situation, and what is he/she doing?,” “How is the person feeling in this situation, and how are his/her feelings for his/her partner?,” and “Why is the person feeling this way?.” Two out of five trained coders were randomly assigned to each case and independently coded the occurrence of communal imagery. Communal content categories included *Emotional Closeness, Positive Evaluations, Empathy, Commitment/Community, Personal Encounters, Attachment*, and *Fear of Loneliness* (for details on the scoring rules, see Hagemeyer and Neyer, 2012). Ambiguous cases were resolved by discussion among all coders. Interrater agreement was high, *ICC*(1, 2) = 0.96. To compute raw motive scores, the number of communal imagery across all eight pictures was summed up and then averaged across the two coders. Because participants' raw motive scores were correlated with the length of the written answers (*r* = 0.41), we residualized the raw motive scores for word count using robust regression techniques as recommended by Schönbrodt et al. (2020).

#### 4.2.3. Experience Sampling: Momentary Communal Behavior

Participants indicated their communal behavior toward their partners on two different experience sampling measures. First, participants described their own behavior since the last survey by tapping on an interpersonal circumplex grid (IPC) presented on the touchscreen of their mobile devices. The IPC grid maps the extent of participants' communal behavior on the x-axis (continuously ranging from 0 = *rejecting* to 1 = *friendly*) and their agentic behavior on the y-axis (continuously ranging from 0 = *unobtrusive* to 1 = *dominant*)^4^. For the present analyses, only the values entered on the communion axis were used. Henceforth, we refer to these scores as *global communal behavior*.

Second, *via* multiple-choice items, participants indicated whether they had displayed one or more of a set of different communal and uncommunal behaviors toward their partners since the last survey. Behavioral options included *affection, admiration, teasing, sacrificing, supporting, asking about feelings and thoughts*, and *paying particularly high regard to the partner*. Uncommunal behaviors included *disinterest or negligence toward the partner* and *paying particularly low regard to the partner*. Each behavioral option was weighted according to its instrumentality for attaining communal end states^5^. The weighted options were then added up to a composite index. Thus, more positive scores of this index indicated more communal behavior, with values ranging from −1 to 4.5. Henceforth, we refer to these index scores as *specific communal behaviors*. The specific communal behaviors were pre-tested in a preceding experience sampling study (Zygar et al., 2018), which demonstrated their positive associations with momentary communal motivation.

#### 4.2.4. Experience Sampling: Perception of Partner's Momentary Communal Behavior

Participants' perceptions of their partners' communal behavior were assessed analogously to their self-rated communal behavior. Participants indicated their partners' communal behavior *via* an IPC grid and by picking one or more from a list of specific (un)communal behaviors. For both measures, answer options and modalities were identical to the measures used for participants' self-rated behavior.

#### 4.2.5. Experience Sampling: Momentary Communal Need Satisfaction

In the current investigation, we operationalized communal need satisfaction as felt closeness to the partner, which lies at the heart of partner-related communion (Hagemeyer and Neyer, 2012). Participants indicated their feelings of closeness to their partners using the pictorial Inclusion of Other in the Self Scale (*IOS*; Aron et al., 1992). Previous studies have demonstrated the good psychometric qualities of this measure and its validity as an indicator of perceived closeness in couple relationships (e.g., Aron et al., 1992, 1997; Agnew et al., 1998). The IOS scale presents seven figures depicting the self and the partner as increasingly overlapping circles. Participants were prompted to select one of these seven pictures in response to the question: “How close (emotionally) do you feel to your partner right now?.” Thus, need satisfaction scores could range from 1 to 7 with higher scores indicating higher satisfaction.

#### 4.2.6. Experience Sampling: Momentary Relationship Satisfaction

Three experience sampling items were used to assess momentary relationship satisfaction: “How are you feeling at the moment in your relationship?” (ranging from 0 = *totally frustrated* to 10 = *totally satisfied*), “How do you feel about your relationship at the moment?” (ranging from 0 = *bad* to 10 = *exceptionally good*), and “How annoyed are you about your partner at the moment?” (reverse-coded; ranging from 0 = *not at all* to 10 = *strongly*). An average score across the three items was computed^6^.

## 5. Results

### 5.1. Descriptive Statistics

Table 2 presents the means and standard deviations of all measures. For the experience sampling measures, means and standard deviations across participants' individual person-means and person-standard deviations are displayed. As detailed in Supplementary Table 1, women listed more specific perceived communal partner behaviors than men (*p* < 0.001). No further significant sex differences were found.

**Table 2:** Descriptive statistics.

	***M*(*SD*) / *M*_Grand_(*SD*)**		***SD*_Grand_(*SD*)**		**Between-person variance (%)**		**Within-person and error variance (%)**	
**Variables**	**Men**	**Women**	**Men**	**Women**	**Men**	**Women**	**Men**	**Women**
pnCommunion	4.95 (2.04)	5.55 (2.03)	–	–	–	–	–	–
Desire for closeness	6.03 (0.66)	6.04 (0.85)	–	–	–	–	–	–
Global communal behavior	7.40 (1.13)	7.41 (1.08)	1.27 (0.44)	1.42 (0.44)	38.69	36.45	61.31	63.55
Perception of partner's global communal behavior	7.30 (1.14)	7.37 (1.15)	1.41 (0.48)	1.49 (0.49)	35.86	36.33	64.14	63.67
Specific communal behaviors	1.24 (0.67)	1.29 (0.61)	0.87 (0.25)	0.90 (0.25)	33.90	29.92	66.10	70.08
Perception of partner's specific communal behaviors	1.14 (0.67)	1.27 (0.62)	0.82 (0.26)	0.89 (0.25)	35.09	31.54	64.91	68.46
Communal need satisfaction	4.43 (1.33)	4.33 (1.26)	1.07 (0.42)	1.20 (0.45)	53.33	50.73	46.67	49.27
Relationship satisfaction	7.87 (1.11)	7.84 (1.12)	1.04 (0.49)	1.14 (0.48)	46.28	46.71	53.72	53.29

*N = up to 60,779 surveys of 508 individuals from 258 couples. M_Grand_, Mean of individual person-means; SD_Grand_, mean of individual person-standard deviations. Between-person variances correspond to intraclass correlations calculated via two-intercept models with random intercepts. Within-person and error variances correspond to the subtraction of the respective intraclass correlation from 1*.

Table 2 also details the relative proportion of between-person variance versus within-person and error variance of the experience sampling measures. These variances were determined by calculating intraclass correlations with the use of unconditional two-intercept models. Between-person variances were substantial (>29%), but mostly lower than within-person and error variances (>46%) for both men and women. This finding suggests that the experience sampling measures assessed, to a large part, moment-to-moment variations (see also Schönbrodt et al., 2021).

### 5.2. Hypothesis Testing

We tested our hypotheses with multilevel modeling to account for the hierarchical structure of the data. The data included up to 140 observations (level 1) for each member (level 2) of a couple (level 3)^7^. Because random variability at level 2 cannot be estimated, we used two-intercept models representing the three conceptual levels of dyadic experience sampling data with two levels (Bolger and Laurenceau, 2013). Two-intercept models specify separate intercepts for male and female partners; the lower level represents within-person variability in male and female partners' repeated measures, and the higher level captures variation in male and female partners' measures across couples. We allowed intercepts to vary randomly across couples (Barr et al., 2013). Effects were likewise modeled as random, but fixed if convergence problems occurred. In all models, we controlled for potential systematic changes in outcomes due to (i) linear trends over time (survey number) and (ii) differences between weekdays and weekends (dummy coded variable: 1 = *weekend*, 0 = *weekday*).

To disentangle within- from between-person associations between outcomes and our level 1 predictor variables, we centered participants' values around their individual person-means. The resulting level 1 variables thus captured pure within-person deviations of the predictors from their typical levels. All multilevel analyses were carried out twice: Once analyzing global (perceived) communal behavior, and once analyzing specific (perceived) communal behavior, thus realizing conceptual replications of the results across two different measures. To account for the increased probability of type I errors, we only considered effects with a *p* < 0.01.

#### 5.2.1. Hypothesis 1: Prediction of Communal Behavior

Hypothesis 1 was tested by means of cross-level interaction models. Actors' momentary communal behavior was regressed on their communal motive dispositions (level 2), their perceptions of their partners' momentary communal behavior (level 1), and the cross-level interaction between the two. We ran a total of four cross-level interaction models to consider actors' explicit as well as implicit communal motive dispositions (explicit desire for closeness and implicit pnCommunion, respectively) and both global and specific (perceived) communal behavior in our analyses.

##### 5.2.1.1. Explicit Desire for Closeness

**Figure 2:**
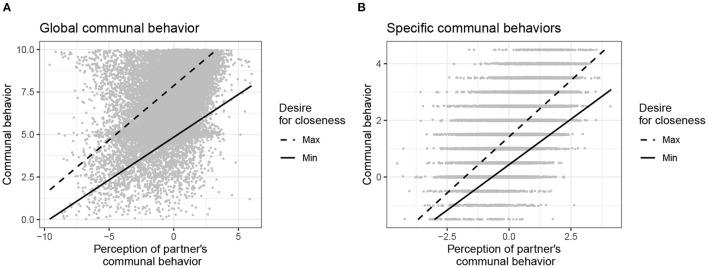
Prediction of communal behavior by the cross-level interaction between individuals' desire for closeness and their perceptions of their partners' communal behavior. **(A)** Global communal behavior. **(B)** Specific communal behaviors. Figure available at: https://osf.io/2fz5w/ under a CC-BY4.0 license.

The upper part of Table 3 details the results of the analyses using actors' explicit desires for closeness as cross-level moderators. Estimates were mostly consistent across the analyses of global and specific communal behavior. Both analyses revealed significantly positive main effects of the explicit desire for closeness. Thus, actors with stronger desires for closeness behaved, on average, more communally than those with weaker desires. Moreover, actors' momentary communal behavior was strongly related to their partner perceptions, meaning that actors engaged in more communal behavior when they perceived their partners to show more communal behavior. Most importantly, there were small but significant interaction effects indicating that actors with stronger desires for closeness were more likely to engage in communal behavior at moments when they rated their partner's behavior as more communal, compared to actors with weaker desires. As illustrated in Figure 2, the positive associations between partner perceptions and actors' own communal behavior increased with the strength of actors' desires for closeness. Simple slopes estimated for maximum and minimum values of the desires for closeness were all significant and positive, both in the analysis of global communal behavior and specific communal behaviors (for details, see Supplementary Table 2).

**Table 3:** Results of multilevel analyses for the prediction of communal behavior by perceptions of partner's communal behavior and communal motives.

		**Global communal behavior**				**Specific communal behaviors**			
**Effects**		**Estimate**	**SE**	**p**	**CI**	**Estimate**	**SE**	**p**	**CI**
Explicit desire for closeness									
	Male intercept	7.352	0.066	<0.001	[7.222; 7.482]	1.227	0.042	<0.001	[1.145; 1.308]
	Female intercept	7.356	0.060	<0.001	[7.239; 7.474]	1.276	0.037	<0.001	[1.204; 1.348]
	Desire for closeness	0.539	0.057	<0.001	[0.427; 0.650]	0.175	0.032	<0.001	[0.112; 0.239]
	Perception of partner's communal behavior	0.616	0.011	<0.001	[0.595; 0.637]	0.768	0.007	<0.001	[0.755; 0.782]
	Desire for closeness × perception of partner's communal behavior	0.024	0.006	<0.001	[0.012; 0.037]	0.027	0.006	<0.001	[0.015; 0.039]
Implicit pnCommunion									
	Male intercept	7.354	0.071	<0.001	[7.215; 7.493]	1.226	0.042	<0.001	[1.143; 1.309]
	Female intercept	7.354	0.067	<0.001	[7.222; 7.486]	1.277	0.039	<0.001	[1.201; 1.353]
	pnCommunion	0.048	0.023	0.041	[0.002; 0.094]	0.010	0.013	0.447	[−0.015; 0.034]
	Perception of partner's communal behavior	0.616	0.011	<0.001	[0.595; 0.637]	0.768	0.007	<0.001	[0.754; 0.782]
	pnCommunion × perception of partner's communal behavior	0.014	0.002	<0.001	[0.010; 0.019]	−0.001	0.002	0.700	[−0.005; 0.003]

*N = up to 51,009 surveys (global communal behavior) and up to 50,949 surveys (specific communal behaviors). Estimate = unstandardized regression coefficients. CI = 95% confidence intervals. Not displayed: effects of covariates weekend and time*.

##### 5.2.1.2. pnCommunion

**Figure 3:**
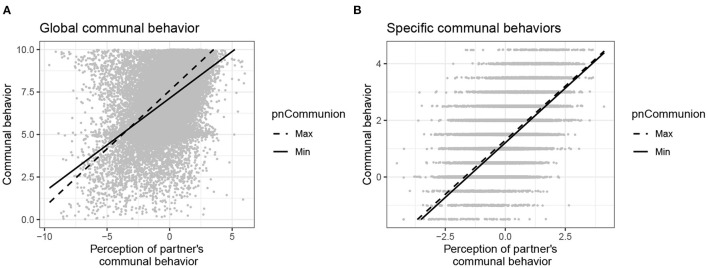
Prediction of communal behavior by the cross-level interaction between individuals' pnCommunion and their perceptions of their partners' communal behavior. **(A)** Global communal behavior. **(B)** Specific communal behaviors. Figure available at: https://osf.io/2fz5w/ under a CC-BY4.0 license.

The results of the analyses using actors' pnCommunion as a cross-level moderator are detailed in the lower part of Table 3. The analyses of global and specific communal behavior yielded a significantly positive main effect of actors' perceptions of their partners' communal behavior, indicating that actors behaved more communally when they perceived their partners' behavior as high in communion. No significant main effect (at the *p* < 0.01 level) of actors' pnCommunion was found. However, in the analysis of global communal behavior, pnCommunion moderated the effect of perceived communal partner behavior. Compared to actors with a weaker pnCommunion, actors with a strong pnCommunion behaved more communally at moments when they perceived their partners to behave highly communally as well (see Figure 3A). Simple slopes estimated for the maximum and minimum pnCommunion values were significantly positive (see Supplementary Table 2). In the analysis of specific communal behaviors, the cross-level interaction effect did not reach statistical significance (see Figure 3B).

#### 5.2.2. Hypothesis 2: Prediction of Communal Need Satisfaction

To address Hypothesis 2, we employed multilevel polynomial regression models with response surface analyses (RSA; Edwards and Parry, 1993; Nestler et al., 2019). In polynomial regression, an outcome is regressed on two predictor variables, their quadratic terms, and their product interaction term. In our models, actors' momentary communal need satisfaction was regressed on their own momentary communal behavior (*b*_1_) and its square (*b*_3_), their perception of their partners' momentary communal behavior (*b*_2_) and its square (*b*_5_), and the interaction between the two linear variables (*b*_4_). In RSA, the two predictor variables should have a common and meaningful zero-point (Schönbrodt et al., 2018; Nestler et al., 2019). Instead of centering the two variables on their individual person-means (as was done for the cross-level interaction models described above), we therefore centered both variables on their pooled person-means. We fitted two polynomial regression models for global and specific communal behavior(s), respectively. As detailed in Table 4, both analyses produced largely convergent results. All polynomial regression coefficients reached statistical significance. Most notably, actors' communal behavior and perceptions of their partners' communal behavior evinced positive main effects and positive interaction effects on communal need satisfaction.

**Table 4:** Results of multilevel response surface analyses for the prediction of communal need satisfaction by communal behavior and perceptions of the partner's communal behavior.

		**Global communal behavior**				**Specific communal behaviors**			
**Effects**		**Estimate**	**SE**	**p**	**CI**	**Estimate**	**SE**	**p**	**CI**
Effects									
	Male intercept	4.330	0.083	<0.001	[4.168; 4.492]	4.389	0.082	<0.001	[4.228; 4.551]
	Female intercept	4.230	0.078	<0.001	[4.076; 4.384]	4.291	0.079	<0.001	[4.137; 4.446]
	Communal behavior (b_1_)	0.171	0.008	<0.001	[0.156; 0.186]	0.196	0.013	<0.001	[0.172; 0.221]
	Perception of partner's communal behavior (b_2_)	0.233	0.008	<0.001	[0.218; 0.248]	0.338	0.013	<0.001	[0.313; 0.364]
	Communal behavior^2^ (b_3_)	−0.011	0.003	<0.001	[−0.017; −0.006]	−0.077	0.010	<0.001	[−0.096; −0.059]
	Communal behavior × perception of partner's communal behavior (b_4_)	0.033	0.003	<0.001	[0.027; 0.039]	0.144	0.015	<0.001	[0.115; 0.173]
	Perception of partner's communal behavior^2^ (b_5_)	−0.016	0.002	<0.001	[−0.020; −0.011]	−0.107	0.010	<0.001	[−0.127; −0.087]
RSA parameters									
	a_1_	0.404	0.006	<0.001	[0.391; 0.416]	0.534	0.009	<0.001	[0.517; 0.552]
	a_2_	0.006	0.002	0.011	[0.001; 0.011]	−0.040	0.007	<0.001	[−0.054; −0.026]
	a_3_	−0.062	0.014	<0.001	[−0.089; −0.034]	−0.142	0.024	<0.001	[−0.189; −0.095]
	a_4_	−0.060	0.006	<0.001	[−0.071; −0.049]	−0.328	0.029	<0.001	[−0.386; −0.271]

*N = 21,892 surveys. Estimate = unstandardized regression coefficients. CI = 95% confidence intervals. Not displayed: effects of covariates weekend and time*.

The polynomial regression coefficients were then used to compute the response surface parameters *a*_1_, *a*_2_, *a*_3_, and *a*_4_, which detail how different combinations of actors' communal behavior with their perceptions of their partners' communal behavior relate to actors' communal need satisfaction^8^. We visualize the resulting response surfaces as three-dimensional plots. The overall shape of a response surface is determined by the linear and quadratic curvature of two lines: The line of congruence (LOC; predictor 1 = predictor 2) and the line of incongruence (LOIC; predictor 1 = −predictor 2). In the present study, a positive linear term coefficient of the LOC (*a*_1_ = *b*_1_+*b*_2_) would signify a positive additive main effect in that actors are more satisfied the higher they rate both their own and their partners' momentary communal behavior. A positive quadratic term coefficient of the LOC (*a*_2_ = *b*_3_+*b*_4_+*b*_5_) would indicate that increases in need satisfaction are stronger in the area of very high values of the two predictor variables. A positive linear term coefficient of the LOIC (*a*_3_ = *b*_1_−*b*_2_) would mean that actors' need satisfaction is higher if they rate their own momentary behavior to be more communal than their partners'; this would reflect in a response surface that is shifted along the LOIC to the lower right corner. Finally, a positive quadratic term coefficient of the LOIC (*a*_4_ = *b*_3_−*b*_4_+*b*_5_) would point to a dissimilarity effect in that higher dissimilarity between self-ratings and partner perceptions is associated with lower need satisfaction. Conversely, this means that higher similarity is associated with higher need satisfaction. All plots feature a black-lined polygon indicating the interpretable region of the response surface (comprising the actual data points excluding outliers; Schönbrodt, 2016).

**Figure 4:**
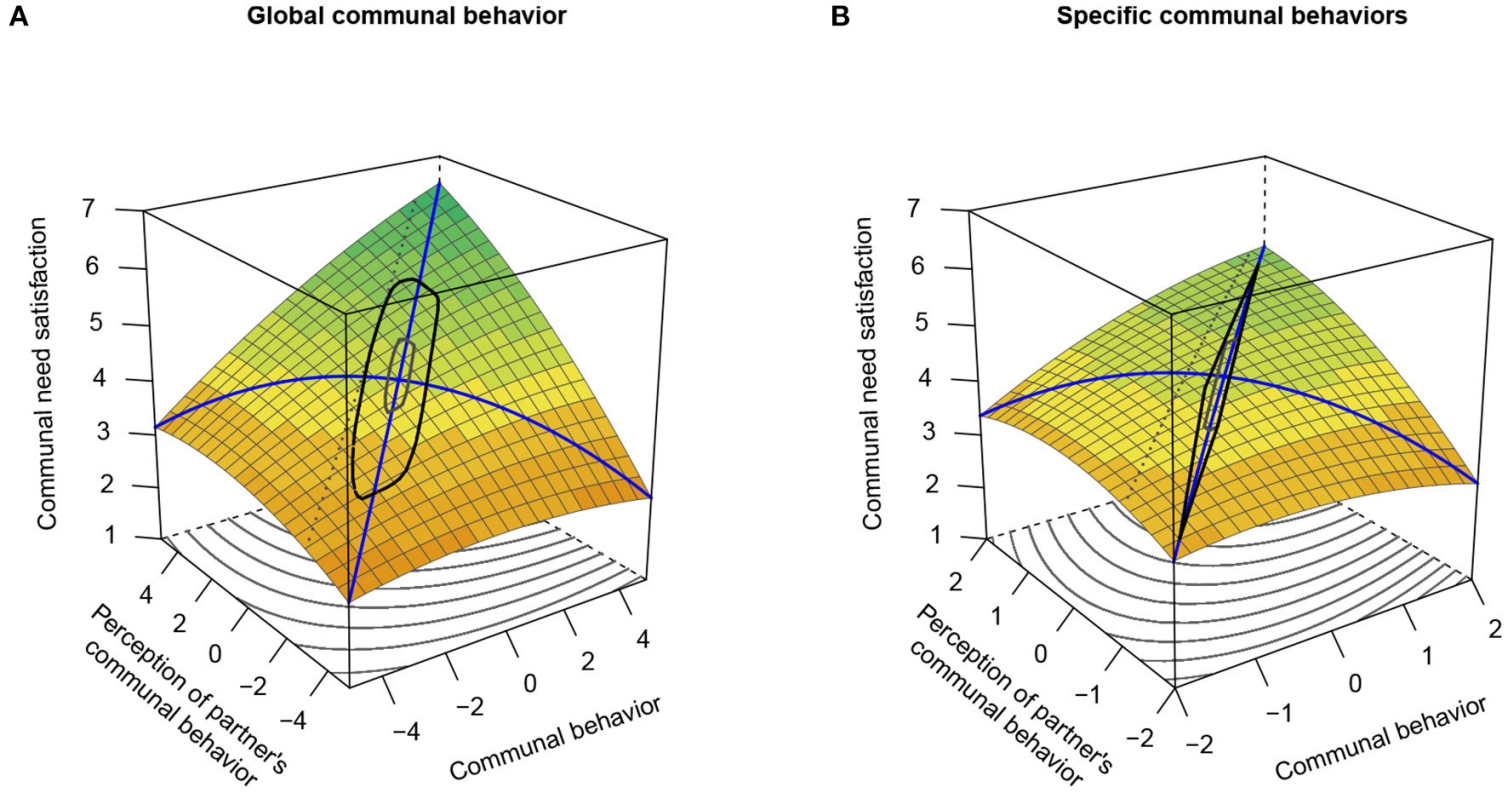
Response surfaces for the prediction of momentary communal need satisfaction by individuals' momentary communal behavior and perceptions of their partners' momentary communal behavior. **(A)** Global communal behavior. **(B)** Specific communal behaviors. Figure available at: https://osf.io/2fz5w/ under a CC-BY4.0 license.

According to Hypothesis 2, we expected that actors' momentary communal behavior and perceptions of their partners' communal behavior would show positive additive main effects as well as positive similarity effects on actors' momentary communal need satisfaction. In our RSAs, this would correspond to a significantly positive *a*_1_ response surface parameter and a significantly negative *a*_4_ response surface parameter, respectively. As shown in Table 4 and illustrated in Figure 4, we found evidence for both. Thus, actors reported more communal need satisfaction at moments when they rated their own and their partners' behavior to be more communal and more similar.

In addition, the LOCs of the response surfaces were also significantly curved (reflected by significant *a*_2_ response surface parameters), albeit in different directions. In the RSA of global communal behavior (Figure 4A), there was a positive quadratic term coefficient of the LOC, indicating that the increase in communal need satisfaction was highest for extremely positive values of both predictors. Concerning the RSA of specific communal behaviors, we found a negative quadratic term coefficient of the LOC. This indicates a plateau of need satisfaction when self- and partner-perceptions of communal behavior were on a relatively high level. However, as can be seen in Figure 4, the areas of extreme (perceived) communal behavior values were cut off from the black-lined polygon comprising the actual data points (excluding outliers). Thus, no firm conclusions regarding the significant *a*_2_ parameters can be drawn, and we refrain from interpreting them. Further, negative *a*_3_ response surface parameters were estimated. Hence, momentary communal need satisfaction was higher when actors rated their partners' behavior as slightly more communal than their own behavior as indicated by a response surface slightly shifted to the lower-left corner (Figure 4).

In sum, both response surfaces indicate that the most beneficial constellation for communal need satisfaction is characterized by actors perceiving themselves and their partners to behave in a similar, highly communal manner, but their partners' behavior as slightly more communal than their own.

However, we note that in the analysis of specific communal behaviors (Figure 4B), the interpretable region (i.e., the region within the black polygon) was relatively narrow. Participants rarely reported pronounced discrepancies between their own and their partners' perceived specific communal behaviors (see Supplementary Figure 1). Thus, although the result pattern matched the RSA of global communal behavior, there was not sufficient data to conclusively support our hypothesis in the analysis of specific communal behaviors.

#### 5.2.3. Hypothesis 3: Prediction of Relationship Satisfaction

To test Hypothesis 3, we estimated the multilevel polynomial regression models described above with actors' momentary relationship satisfaction as the outcome variable. We again fitted two models analyzing (perceived) global and specific communal behavior, respectively. The results are presented in Table 5. In both analyses, all polynomial effects reached statistical significance, including the focal interaction between actors' communal behavior and perceptions of their partners' communal behavior.

**Table 5:** Results of multilevel response surface analyses for the prediction of relationship satisfaction by communal behavior and perceptions of the partner's communal behavior.

		**Global communal behavior**				**Specific communal behaviors**			
**Effects**		**Estimate**	**SE**	**p**	**CI**	**Estimate**	**SE**	**p**	**CI**
Effects									
	Male intercept	8.030	0.067	<0.001	[7.898; 8.162]	8.019	0.069	<0.001	[7.883; 8.154]
	Female intercept	8.027	0.066	<0.001	[7.897; 8.156]	8.001	0.069	<0.001	[7.865; 8.136]
	Communal behavior	0.165	0.005	<0.001	[0.156; 0.174]	0.107	0.008	<0.001	[0.090; 0.123]
	Perception of partner's communal behavior	0.248	0.005	<0.001	[0.239; 0.257]	0.365	0.009	<0.001	[0.348; 0.382]
	Communal behavior^2^	−0.038	0.002	<0.001	[−0.041; −0.035]	−0.112	0.007	<0.001	[−0.125; −0.100]
	Communal behavior × perception of partner's communal behavior	0.046	0.002	<0.001	[0.043; 0.050]	0.213	0.010	<0.001	[0.193; 0.233]
	Perception of partner's communal behavior^2^	−0.054	0.001	<0.001	[−0.057; −0.052]	−0.200	0.007	<0.001	[−0.213; −0.186]
RSA parameters									
	a_1_	0.413	0.004	<0.001	[0.406; 0.421]	0.472	0.006	<0.001	[0.460; 0.483]
	a_2_	−0.046	0.001	<0.001	[−0.049; −0.043]	−0.100	0.005	<0.001	[−0.109; −0.090]
	a_3_	−0.083	0.008	<0.001	[−0.100; −0.067]	−0.259	0.016	<0.001	[−0.290; −0.228]
	a_4_	−0.139	0.003	<0.001	[−0.146; −0.132]	−0.525	0.020	<0.001	[−0.564; −0.486]

*N = 51,009 surveys (global communal behavior) and 50,949 (specific communal behaviors). Estimate = unstandardized regression coefficients. CI = 95% confidence intervals. Not displayed: effects of covariates weekend and time*.

**Figure 5:**
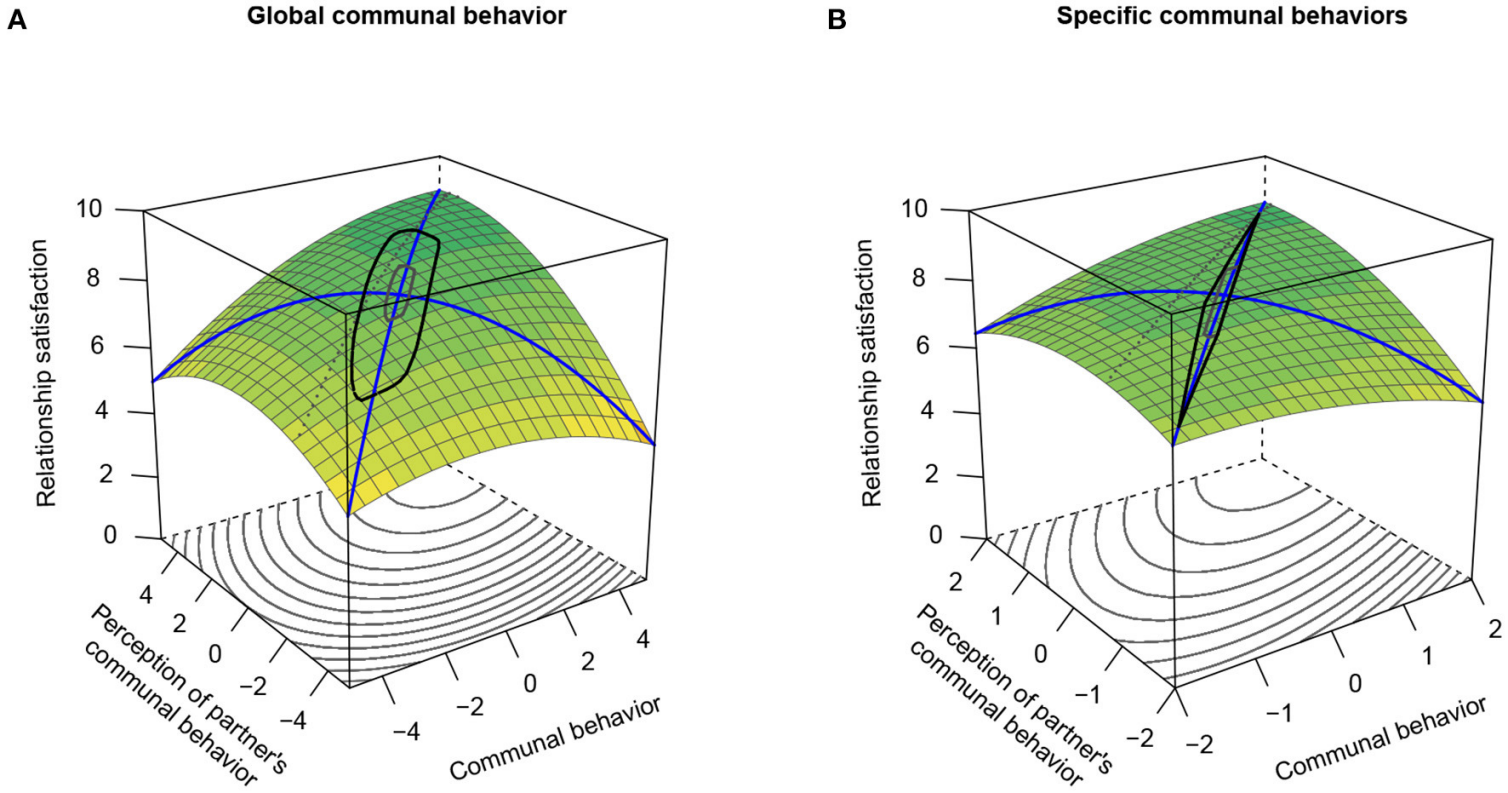
Response surfaces for the prediction of momentary relationship satisfaction by individuals' momentary communal behavior and perceptions of their partners' momentary communal behavior. **(A)** Global communal behavior. **(B)** Specific communal behaviors. Figure available at: https://osf.io/2fz5w/ under a CC-BY4.0 license.

These findings reflect in the corresponding response surfaces illustrated in Figure 5. As expected, we found significantly positive *a*_1_ response surface parameters and significantly negative *a*_4_ response surface parameters across both operationalizations of (perceived) communal behavior. Thus, actors' communal behavior and perceptions of their partners' communal behavior evinced positive additive main effects and positive similarity effects on individuals' momentary relationship satisfaction. In other words, actors reported higher relationship satisfaction when they rated their own and their partners' behavior as more communal and more similar.

Further, both response surfaces show a negative quadratic term coefficient of the LOC (negative *a*_2_ response surface parameters). This indicates that relationship satisfaction reached a plateau when self- and partner-perceptions of communal behavior were on a relatively high level. Moreover, the response surface showed a shift to the lower-left corner (negative *a*_3_ response surface parameters), meaning that actors were more satisfied when they perceived their partners' behavior to be slightly more communal than their own behavior.

To summarize, both response surfaces suggest that actors are most satisfied with their relationship if they perceive their own and their partners' behavior as highly communal and largely similar, but their partners' behavior as slightly more communal than their own. Again, this result pattern underlines the relevance of perceived behavioral similarity for relationship satisfaction, albeit not according to strict conceptualizations of similarity (Humberg et al., 2018).

Again, however, there was not sufficient evidence for the additive and similarity effects of (perceived) specific communal behaviors, as the interpretable region of the response surface (Figure 5B) was quite narrow^9^.

### 5.3. Supplemental Analyses

The MIC model states that the partner's communal behavior can only contribute the actor's motivation if it is subjectively perceived as a cue (*Motivational Cues* implication in Table 1). To corroborate this assumption, we additionally examined whether partners' self-rated communal behavior bears motivational relevance in its own right. Although the partner's self-rated behavior is no perfect indicator of the partner's rather objective, actual behavior, it may nonetheless capture certain aspects of the partner's behavior not accessible to the actor (Vazire, 2010). We therefore repeated all main analyses using partners' self-rated (instead of perceived) communal behavior as a predictor.

First, there was no robust evidence that partners' self-rated behavior contributed to actors' communal motive expression (see Supplementary Table 3). Although partners' self-rated communal behavior showed significantly positive effects on actors' communal behavior, their interactions with actors' communal motives made no significant contributions to the prediction. Moreover, all main results pertaining to Hypothesis 1 were robust when controlling for partners' self-rated communal behavior and its interaction with actors' motives (see Supplementary Table 4). Thus, it appears that perceived communal partner behavior is more relevant for actors' communal motive expression than partners' self-rated behavior.

Second, we found evidence that partners' self-rated behavior contributes to actors' communal motive satisfaction (see Supplementary Tables 5, 7). In supplemental RSAs, actors' and their partners' self-rated communal behavior showed significant additive main effects (reflected by positive *a*_1_ response surface parameters) as well as significant similarity effects (reflected by negative *a*_4_ response surface parameters). The exception was the prediction of communal need satisfaction by specific communal behaviors (Supplementary Table 5), where no significant similarity effect was found. Further, the effects of perceived behavior similarity remained robust: Even when controlling for partners' self-rated communal behavior (and its higher-order interactions), the focal additive main effects (positive *a*_1_ response surface parameters) and similarity effects (negative *a*_4_ response surface parameters) of actors' own communal behavior and perceptions of their partners' communal behavior remained significant (see Supplementary Tables 6, 8). All in all, these results suggest that actors' need satisfaction and relationship satisfaction are higher at moments when they perceive their own and their partners' behavior as more communal and more similar as well as at moments when couple members' self-rated behavior is more communal and more similar.

## 6. Discussion

This article presented an integrative model of interdependence between couple members' motive dispositions. The MIC model proposes that couple members provide situational cues for each other's motivation through their observable motivated behavior. Interpersonal perception constitutes the gateway for these motivational transactions. The partner's motivated behavior is proposed to affect the actor's motivation only if the actor perceives it as linked with motive-specific incentives. In this regard, the MIC model proposes a functional motivational explanation of how and when partner influences can become psychologically meaningful.

Data from an extensive experience sampling study provided support for the two core assumptions of the MIC model. Applying the MIC model to partner-related communion motivation, we hypothesized that actors' subjective perceptions of their partners' momentary communal behavior would contribute to actors' (i) communal motive expression and (ii) communal motive satisfaction. In the following, we discuss how the results relate to our expectations. We then turn to study limitations before addressing broader implications of the MIC model for existing theories and future research.

### 6.1. Motive Expression in Couple Relationships

The results of our cross-level interaction models indicate that actors behaved more communally when they perceived their partners' momentary behavior as more communal. In support of Hypothesis 1 and the Motive Expression assumption (see Table 1), this association was even stronger for actors with stronger explicit or implicit communal motives. This finding suggests that perceived partner behavior can arouse actors' motives and thereby contribute to the instigation of actors' motivated behavior. Although motivation research knows numerous examples of other persons' behavior functioning as motivational cues (e.g., Atkinson et al., 1954; Fodor and Wick, 2009; Hagemeyer et al., 2016), a direct investigation of this idea in the context of everyday couple interactions has been lacking. The present analyses suggest that partner behavior can likewise motivate actor's own behavior, but—in line with the Motivational Cues implication (see Table 1)—only if it is subjectively perceived as motivationally relevant.

These findings contribute to a deeper understanding of how motive dispositions express in partner-related behavior. Numerous studies have shown that two partners tend to reciprocate each other's communal behavior (Sadler and Woody, 2003; Markey and Markey, 2007; Sadler et al., 2009; Markey et al., 2010; Dermody et al., 2017), which seems to foster need satisfaction on both sides (Horowitz et al., 2006). The current findings emphasize that as a potential motivational cue promising the attainment of motive-specific incentives, partners' interpersonal behavior can arouse each other's motivated behavior. According to the MIC model, this process largely depends on individual motive strength. Motive strength should not only guide how actors perceive their partners' behavior, but also determine how readily actors react to their partners with complementary behavior.

Further, the current findings add to existing evidence of the relevance of both implicit and explicit motives for relationship functioning (e.g., Hagemeyer et al., 2013a; Zygar-Hoffmann et al., 2020b). In the current investigation, both implicit and explicit communal motives predicted communal behavior in interaction with perceived communal partner behavior. The interaction effects of implicit communal motives were less robust however, likely because propositional partner perceptions, as employed in the current study, have stronger conceptual links with explicit motives (McClelland et al., 1989). Future studies should therefore consider additional, non-propositional indicators of partner perception (e.g., implicit partner evaluations; Fazio and Olson, 2003), which should more likely function as motivational cues for implicit motives. Similarly, future research may use more objective indicators of behavior (e.g., behavior observations) that can also capture nonverbal types of behavior (e.g., expressions of emotion; McClelland, 1987; Schultheiss, 2001). We expect that complementary analyses of measures that are better aligned with implicit motives will further substantiate the found associations.

All in all, the effects of the cross-level interactions between communal motives and partner perceptions were small and not robust in supplemental analyses of the smaller-sized preceding study sample. They thus require replication with high-powered independent data. Nonetheless, small effects can have meaningful consequences (Funder and Ozer, 2019; Götz et al., 2021). Even a slightly higher tendency to engage in communal behavior may facilitate smoother interactions between partners on numerous occasions. In the long run, such short-term benefits may accumulate and considerably benefit overall relationship functioning. In a similar vein, the large main effects of perceived partner behavior, albeit very likely inflated by shared method variance, suggest that other mechanisms unrelated to interindividual differences in motive dispositions might be at play too. The MIC model does not disclaim such main effects. For example, individuals might normatively expect their partners to reciprocate their own communal behavior, or couples might develop largely habitual interaction patterns over the course of their relationship. Although such interaction patterns can be based on earlier motivational processes, they constitute rather automatic behavioral scripts that do not need to be initiated by current motivational impulses. Hence, further research is needed to investigate the development of motivational interdependence patterns over the course of romantic relationships.

### 6.2. Motive Satisfaction in Couple Relationships

Our findings provide support for Hypotheses 2 and 3, which focused on the *Motive Satisfaction* implication of the MIC model (Table 1). As expected, engaging in communal behavior was positively related to actors' momentary communal need satisfaction and relationship satisfaction, particularly when actors' perceived their partners to show similarly strong communal behavior at the same time. Conversely, perceiving their own and their partners' behavior as too divergent was associated with need frustration and low satisfaction. Actors' perceptions of their partners' behavior thus appear to function as either opportunities or barriers for incentive attainment. These results agree with previous research showing that perceptions of a responsive and need-supportive partner can foster incentive attainment and satisfaction (e.g., Brunstein et al., 1996; Lemay et al., 2007), whereas perceiving the partner as unsupportive seems to drive dissatisfaction and conflicts (Vanhee et al., 2016a,b; Petit et al., 2017). The current results present an important addition to this literature by showing how these associations come about on the intrapersonal level. Importantly, our analyses showed that the proposed mechanism of motive satisfaction not only explains fluctuations in communal need satisfaction, but also in relationship satisfaction. Relationship satisfaction is perhaps the most investigated indicator of relationship functioning. Thus, the current results provide additional support for the relevance of the MIC model for understanding relationship functioning more broadly, beyond purely motivational outcomes.

Interestingly, the results of our RSAs indicate that perceiving the partner's behavior as slightly more communal than one's own behavior further adds to satisfaction, even beyond perfect perceived similarity. Hence, perceived partner behavior that slightly exceeds one's own communal behavior seems particularly rewarding. Probably, such perceptions signal that the partner is genuinely interested in communion, and not only acts out of habit or normative demands. This idea agrees with recent research (Zoppolat et al., 2020) suggesting that perceived sacrifices by the partner (such as putting self-interests aside for the sake of the relationship) are experienced as more pleasant by individuals who do not expect such sacrifices, compared to those who consider sacrifices as rather normal and necessary (see also Visserman et al., 2018). However, additional research is needed to substantiate this *post-hoc* explanation.

In most experience sampling surveys, participants listed a comparable amount of specific communal behaviors they showed and perceived their partners to show. Discrepancies in specific communal behaviors were rarely reported, which considerably limited the interpretability of the corresponding RSAs: Although the interpretable region of the response surfaces indicated additive main effects of (perceived) specific communal behaviors on satisfaction, there was not sufficient data to support a similarity effect. Nevertheless, the parameter estimates largely converged with those found in the analysis of global communal behavior. Likely, future studies assessing a list of more distinct specific communal behaviors will provide more conclusive evidence for similarity effects.

### 6.3. Limitations and Future Research

Due to the correlational nature of our analyses, our findings do not allow firm conclusions regarding causal associations. Nonetheless, the hypothesized associations were closely derived from theoretical considerations and empirical findings, and our analyses substantiate that everyday within-couple interdependence processes contribute to motivated behavior and need satisfaction. Although such naturalistic studies come with the limitations of any correlational analysis, it is, in our view, an essential first step to examine the mechanisms of motive expression and satisfaction in couples' daily lives. Our findings show that the postulated mechanisms of motive expression and satisfaction matter in the complex dynamics of real-world couple interactions. We expect that future investigations of the short-term dynamics of couples' motivational processes will provide converging evidence. For example, replicating our findings in experimental and observational studies in the laboratory can enhance causal inference.

Moreover, our analyses heavily relied on self-report data, which may have led to biased results due to shared method variance. Especially the strong main effects on self-reported behavior and satisfaction are likely exaggerated. The interaction effects, which are focal to our hypotheses, are less likely to be affected, as shared method variance would rather decrease the chances of finding significant interaction effects (Siemsen et al., 2010). Some found effects, however, may also be underestimated because of the potentially reduced reliability of our single-item measures (Schönbrodt et al., 2021). Future research should replicate our analyses with more objective indicators of individuals' motivated behavior and satisfaction. For example, previous studies suggest that intra-individual motivational processes come along with physiological (e.g., Dufner et al., 2015) and hormonal changes (e.g., Schultheiss et al., 2004). Future research could also utilize the increasing technological possibilities to obtain more objective information about couples' interactions (such as spatial proximity to the partner or the frequency of digitally mediated contact; Harari et al., 2016).

In addition, most participants of the current study were in their young or middle adulthood, well-educated, and from a Western cultural context. This may limit the generalizability of our findings to individuals from other backgrounds. Recent research suggests that socioeconomic context plays an important role in how partners interact with each other and how they express their emotions to each other (Cho et al., 2020; Karney and Bradbury, 2020). More broadly, the cultural context may influence how motives develop (e.g., *via* culture-specific normative expectations, environmental pressures and demands) and which cues and instrumental behaviors people link with motive-specific incentives (McClelland et al., 1989; Hofer, 2010). Thus, additional studies with couples from more diverse social, educational, and occupational backgrounds, life circumstances, and age groups are needed to further substantiate the viability and scope of the MIC model.

The degree of interdependence between couple members is usually stronger than between friends, family members, or work colleagues (Berscheid et al., 1989; Rusbult and Lange, 2003). However, this is not to say that motivational interdependence does not occur in other types of relationships. For example, much like in couple relationships, to feel supported and cared for in a friendship (a communal incentive), the friend needs to be (perceived as) responsive to one's own needs and wishes. Thus, although the MIC model focuses on couple relationships, applying it to other types of relationships is a compelling task for future research.

Finally, future theorizing and research may incorporate additional processes to extend the MIC model. Attachment-related processes, for instance, may help to understand how people perceive and respond to their partner's motivated behavior (Hazan and Shaver, 1994; Velotti et al., 2011). Similarly, investigating how people regulate their emotions may help to better understand how motivationally driven couple interactions contribute to experiences of satisfaction or frustration, and how people handle such experiences (e.g., Rusu et al., 2018).

### 6.4. Broader Implications of the MIC Model

#### 6.4.1. Relations of the MIC Model With Existing Theories

The MIC model is strongly inspired by interdependence theory (IT; Thibaut and Kelley, 1959; Kelley and Thibaut, 1978; Rusbult and Arriaga, 1997). Like the MIC model, IT posits that couple members' behavior and experiences fundamentally depend on and influence each other. IT focuses on the structural aspects of interdependent situations such as the degree and types of interdependence and what people make of interdependent situations (i.e., how readily they transform their self-interests for the sake of their partners). The strength of IT is that it provides a broad framework applicable to various partner attributes. However, due to this breadth, IT cannot fully account for the functional mechanisms of interdependence specific to the attribute under study. Based on core assumptions in motivation psychology, the MIC model explains interdependence between couple members' motives by their behavioral and perceptual functions. This functional approach enables precise predictions of when couple members' motives can affect each other's outcomes (the partner's motivational strivings have to be perceived as complementary to one's own strivings), and how (by contributing to the motivation of behavior and need satisfaction during the motive expression and satisfaction phases, respectively). The MIC model thus complements IT by detailing the functional mechanisms and processes underlying interdependence between couple members' motive dispositions.

The MIC model also shares similarities with the recent transactive goal dynamics theory (TGD; Fitzsimons et al., 2015). TGD proposes that partners can influence each other's goal pursuits to the extent that they form a single unit of self-regulation with shared goals and resources to attain these goals (Fitzsimons et al., 2015). Relationship quality is supposed to largely depend on couple members' goal coordination, i.e., the extent to which their individual goals and goal-pursuit behavior are *compatible*. The MIC model zooms in on this link by elucidating the concept of goal-compatibility and its motivational consequences. Specifically, the MIC model states (i) that compatibility is determined by the perceived complementarity between one's own and the partner's motivational strivings and (ii) that perceived complementarity can promote relationship quality by fueling motivated behavior and need satisfaction. Complementarity, motivated behavior, and need satisfaction can look differently for different types of motives (see Section “Applications of the MIC Model to Partner-Related Motives in the Introduction). The MIC model can thus help to answer TGD's “… neglected questions about how different types or domains of goals … may elicit different types of interdependent patterns” (Fitzsimons et al., 2015, p. 666). Moreover, the MIC model is not limited to explicit goals, but can also be applied to implicitly represented motives.

In a similar vein, the Michelangelo model (Rusbult et al., 2009) states that people can support their partners in progressing toward their ideal-self goals by perceptions and behaviors that affirm these ideals. The MIC model offers a new perspective on the causes and consequences of partner affirmation. From a motivational perspective, the actor needs to be motivated to affirm the partner, for instance, due to having complementary self-ideal goals. Affirmation behavior, in turn, should provide important motivational cues for the partner that contribute to the expression and satisfaction of the partner's self-ideal goals.

Various other models of motivation can be integrated into the MIC model as well. The *Dynamics of Motive Satisfaction model* (Zygar et al., 2018), for instance, adds that motive dispositions express in motivated behavior *via* motivational states (i.e., the current need to attain a motivational incentive) and that motivational states amplify the affective reactions to need satisfying experiences. Other theories detail how people implement their motivation into behavior (Heckhausen and Gollwitzer, 1987; Gollwitzer and Brandstätter, 1997; Gollwitzer, 1999), and how they deal with intra-personal goal conflicts (Riediger and Freund, 2004). Researchers have also argued that experiences of need satisfaction can feed back into individuals' motivational states (e.g., saturation or discrepancy effects) as well as perceptions of their (social) surroundings (Bischof, 1975; Carver and Scheier, 1998). Such elements of the intrapersonal motivational process can easily be added to the MIC model by zooming in on the respective model element. However, the MIC model does not specify them explicitly, not least to remain parsimonious. Purely intrapersonal motivational manifestations should have little relevance for couples' motivational interdependence because they are unobservable from the outside (Mund et al., 2016). Consequently, the MIC model focuses on the interpersonal and observable behavioral manifestations of partners' motivation and their implications for need satisfaction.

#### 6.4.2. New Insights Into Couple Dynamics

The MIC model predicts that people can create motivational opportunities out of their own motives. By shaping their perceived relationship environment in ways that fit their motives (see *Accuracy and Bias* implication in Table 1), individuals may find better conditions to enact these very motives. In this way, biased partner perception may allow individuals to continually make satisfying experiences in their relationship without having to assess their partners' contribution to these experiences every time anew. It appears that biased perception can provide motivational benefits even (or particularly) when times are less rosy (Murray, 1999). For example, if the partner prefers individual activities (e.g., hobbies, meeting friends, working) over shared couple activities, staying convinced of the partner's communal interests may mitigate feelings of loneliness and rejection. This idea agrees with research suggesting that people with strong communal approach motives tend to avoid perceptions of particularly uncommunal partner behavior (Pusch et al., 2020). This may add to explanations of why some people maintain their relationships even when they are deeply unhappy. From the perspective of the MIC model, the intention to maintain the relationship should depend on the (un)realistic conviction that the partner provides means to fulfill certain needs which would be lost if the relationship were to end. Continually perceiving the partner in a motivationally biased way may, in the long run, even change the partner's self-view. The Michelangelo phenomenon (Rusbult et al., 2009) builds on interdependence theory and states that people can help their partners in reaching their ideal-self goals (e.g., obtaining certain skills or resources) by affirming these ideals - either by perceiving their partners or behaving toward their partners in ways that match their partners' ideals. The MIC model helps to better understand such affirmation processes from a motivational perspective. By consistently perceiving the partner in a biased, ideal-congruent way and behaving accordingly, the actor can provide important motivational opportunities for the partner to express and satisfy ideal-self goals. However, the MIC model proposes that for actors to do so, they need to have complementary self-ideal goals.

In a similar vein, the MIC model can be used to identify important sources of couple conflicts. Conflicts may arise if one or both couple members' motive expression or motive satisfaction are hindered. This can not only result from incomplementary motives, but also from misunderstandings and misperceptions. For instance, people may perceive it as indicative of a weak interest in communion if their partner works late often, although there might be other reasons for this behavior (such as a high workload). Indeed, many conflicts between partners arise from overly rigid interpretations of the partner's dispositional motives, in the sense that negative qualities are exaggerated and positive ones downplayed (Gottman and Notarius, 2002). Moreover, conflicts may arise when partners strive to express and satisfy different motives. For example, when a partner frequently seeks proximity in order to feel close (i.e., a communal incentive), the actor may perceive this as clinging and a threat to the pursuit of individual interests and activities (i.e., agentic incentives)—which can lead them to feel constricted and suffocated (Mashek and Sherman, 2004). Couples can develop various strategies to promote both members' wellbeing and need satisfaction. For instance, an open communication of behavioral intentions may prevent misinterpretations of certain behaviors as motivational barriers. Also, couple members may make an effort to pay more attention to and recall each others' motivationally rewarding acts, or find less threatening (and oftentimes more plausible) explanations for each other's behavior. These strategies can be trained, for instance, in the context of couple counseling, demonstrating potential practical applications of the MIC model.

## 7. Conclusion

This paper introduced a functional model of motivational interdependence in couples that integrates theoretical assumptions based on general tenets about motivation, personality, and interpersonal perception. The MIC model argues that couple members' subjective perceptions of each other are central for understanding how they coordinate and negotiate the expression and satisfaction of their individual needs. An extensive experience sampling study on explicit and implicit communal motives provided first empirical support for the MIC model: Perceiving the partner's communal behavior as complementary to one's own communal strivings seems to (i) contribute to the expression of communal motives into communal behavior and (ii) provide opportunities for communal need satisfaction. We hope the MIC model will inspire additional research aimed at unraveling the motivational mechanisms and consequences of interdependence in couple relationships.

## Data Availability Statement

Publicly available datasets were analyzed in this study. This data can be found at: https://doi.org/10.5160/psychdata.zrce18mo99.

## Ethics Statement

The studies involving human participants were reviewed and approved by the Ethic Commission of Friedrich Schiller University Jena. The patients/participants provided their written informed consent to participate in this study.

## Author Contributions

SP, FS, CZ-H, and BH contributed to conception and design of the study. Data were collected by CZ-H and prepared by SP and CZ-H. SP performed the statistical analyses and wrote the manuscript. All authors contributed to manuscript revision, read, and approved the submitted version.

## Funding

This research was funded by grants from the German Research Foundation to BH (HA 6884/2-1; HA 6884/2-2) and FS (SCHO 1334/5-1).

## Conflict of Interest

The authors declare that the research was conducted in the absence of any commercial or financial relationships that could be construed as a potential conflict of interest.

## Publisher's Note

All claims expressed in this article are solely those of the authors and do not necessarily represent those of their affiliated organizations, or those of the publisher, the editors and the reviewers. Any product that may be evaluated in this article, or claim that may be made by its manufacturer, is not guaranteed or endorsed by the publisher.

## Footnotes

^1^Throughout this article, we use the terms “couple” and “partner” to reflect romantic couples and partners, respectively.

^2^Motivational cues must not be mistaken with the common understanding of cues in research on personality perception (e.g., Brunswik, 1956; Funder, 1995; Back et al., 2011; Rauthmann et al., 2015). In this literature, the term *cue* is used to describe objective situational stimuli, whereas psychologically meaningful stimuli are referred to as *characteristics*. Situation characteristics are conceptually similar to what motivation research (and the current paper) refers to as cues, i.e., situational stimuli that possess subjective meaning for the person's motivation.

^3^Version numbers of the R packages are detailed at https://osf.io/2fz5w/.

^4^See https://osf.io/2fz5w/ for a screenshot.

^5^The weights for the specific communal behaviors were determined based on discussion among the four authors. These weights were initially preregistered for a preceding experience sampling study (see the preregistration at https://osf.io/jbuxh/). The preregistration for the current study proposes different weights (see https://osf.io/fhtw5/) and lists additional dyadic behaviors (such as disputes between partners) to choose from that were only included in the behavior indices if actors indicated that the behavior was initiated by themselves or by both themselves and their partners. For the current analyses, we used the weights preregistered for the preceding study and omitted the dyadic behaviors, because we wanted to clearly separate actors' from their partners' (perceived) behavior to examine their unique role for couples' motivational interdependence as proposed by the MIC model.

^6^Using the same data as the current article, Schönbrodt et al. (2021) recently estimated the reliability of the relationship satisfaction scale on a within-person/between-moments level, which amount to 0.58 (Schönbrodt et al., 2021). We are not aware of any established method for the estimation of the within-person reliability of our single-item measures.

^7^The data would additionally allow to account for the day-level (Schönbrodt et al., 2021). In the current study, we did not model a day level because we were interested in moment-to-moment associations.

^8^To compute the response surface parameters, we used an R-Script provided by Nestler et al. (2019), accessible at https://osf.io/jhyu9/.

^9^The current study was preceded by an experience sampling study with 130 individuals from 68 couples. For the sake of transparency and to further substantiate our main findings, we repeated all analyses based on the data of this preceding study. Information on the preceding study and analysis results are detailed in the Supplemental Materials (Section Preceding Experience Sampling Study).
