# Supplementary material for: Motivational Interdependence in Couple Relationships
Source: Front Psychol. 2022 May 23;13:827746. doi: 10.3389/fpsyg.2022.827746 (PMC9169053; doi:10.3389/fpsyg.2022.827746)
Supplement: Supplementary file 1 [file Data_Sheet_1.PDF]

Table S1

Sex Differences in Study Variables

Variables	$t(df)$	p	d
pnCommunion	1.692 (248)	.092	.110
Desire for closeness	0.380 (249)	.704	.013
Global communal behavior	0.165 (249)	.869	.012
Perception of partner's global communal behavior	0.867 (249)	.387	.059
Specific communal behaviors	1.922 (249)	.056	.128
Perception of partner's specific communal behaviors	3.969 (249)	<.001	.256
Communal need relsatisfaction	−1.454 (249)	.147	-.083
Relationship relsatisfaction	−0.500 (249)	.618	-.028

Note. N = between 249 and 250 couples. t-statistics pertain to paired-sample t-tests comparing women's with men's scores. Participants' pnCommunion scores were residualized for word count.

Table S2

Simple Slopes Analysis

	Global communal behavior			Specific communal behaviors		
Effects	Est.	*SE*	CI	Est.	*SE*	CI
**Moderator: Desire for closeness**						
Max	0.639	0.012	[0.615; 0.663]	0.795	0.009	[0.777; 0.812]
Min	0.503	0.032	[0.440; 0.565]	0.642	0.029	[0.586; 0.698]
**Moderator: pnCommunion**						
Max	0.686	0.016	[0.655; 0.716]	0.764	0.012	[0.740; 0.788]
Min	0.550	0.015	[0.520; 0.580]	0.771	0.012	[0.748; 0.795]

Note. N = 51,009 surveys (global communal behavior) and 50,949 surveys (specific communal behaviors). Est. = unstandardized regression coefficients. CI = 95% confidence intervals. *Max* and *Min* denote the maximum and minimum values, respectively, of the motive.

Table S3

Results of Multilevel Analyses for the Prediction of Communal Behavior by Communal Motives and Partner's Self-Rated Communal Behavior

	Global communal behavior				Specific communal behaviors			
Effects	Estimate	*SE*	*p*	CI	Estimate	*SE*	*p*	CI
**Explicit desire for closeness**								
Male intercept	7.359	0.066	<.001	[7.230; 7.488]	1.216	0.042	<.001	[1.133; 1.299]
Female intercept	7.338	0.061	<.001	[7.218; 7.458]	1.262	0.039	<.001	[1.185; 1.338]
Desire for closeness	0.533	0.058	<.001	[0.419; 0.646]	0.173	0.035	<.001	[0.105; 0.240]
Partner's communal behavior	0.202	0.011	<.001	[0.180; 0.224]	0.265	0.010	<.001	[0.246; 0.285]
Desire for closeness × partner's communal behavior	-0.002	0.010	.828	[-0.021; 0.017]	0.009	0.009	.350	[-0.010; 0.027]
**Implicit pnCommunion**								
Male intercept	7.361	0.071	<.001	[7.222; 7.501]	1.216	0.043	<.001	[1.132; 1.300]
Female intercept	7.340	0.068	<.001	[7.206; 7.473]	1.263	0.041	<.001	[1.183; 1.343]
pnCommunion	0.048	0.023	.037	[0.003; 0.094]	0.015	0.013	.237	[-0.010; 0.041]
Partner's communal behavior	0.202	0.011	<.001	[0.180; 0.224]	0.266	0.010	<.001	[0.246; 0.285]
pnCommunion × partner's communal behavior	-0.005	0.004	.160	[-0.012; 0.002]	-0.005	0.003	.094	[-0.012; 0.001]

Note. N = up to 40,928 surveys (global communal behavior) and up to 40,866 surveys (specific communal behaviors). Estimate = unstandardized regression coefficients. CI = 95% confidence intervals. Not displayed: effects of covariates weekend and time.

Table S4

Results of Multilevel Analyses for the Prediction of Communal Behavior by Communal Motives, Perceptions of Partner's Communal Behavior, and Partner's Self-Rated Communal Behavior

	Global communal behavior				Specific communal behaviors			
Effects	Estimate	*SE*	*p*	CI	Estimate	*SE*	*p*	CI
**Explicit desire for closeness**								
Male intercept	7.390	0.066	<.001	[7.261; 7.520]	1.267	0.042	<.001	[1.185; 1.349]
Female intercept	7.381	0.061	<.001	[7.262; 7.500]	1.304	0.038	<.001	[1.230; 1.377]
Desire for closeness	0.533	0.058	<.001	[0.419; 0.646]	0.172	0.033	<.001	[0.106; 0.237]
Perception of partner's communal behavior	0.586	0.004	<.001	[0.579; 0.593]	0.751	0.004	<.001	[0.744; 0.758]
Partner's self-rated communal behavior	0.039	0.004	<.001	[0.031; 0.047]	0.052	0.004	<.001	[0.045; 0.059]
Desire for closeness × Perception of partner's communal behavior	0.047	0.005	<.001	[0.038; 0.057]	0.039	0.005	<.001	[0.029; 0.049]
Desire for closeness × Partner's self-rated communal behavior	-0.019	0.005	.001	[-0.030; -0.008]	-0.013	0.005	.007	[-0.023; -0.004]
**Implicit pnCommunion**								
Male intercept	7.391	0.071	<.001	[7.251; 7.531]	1.266	0.042	<.001	[1.183; 1.349]
Female intercept	7.381	0.068	<.001	[7.248; 7.513]	1.305	0.039	<.001	[1.228; 1.382]
pnCommunion	0.047	0.023	.043	[0.002; 0.093]	0.012	0.013	.322	[-0.012; 0.037]
Perception of partner's communal behavior	0.586	0.004	<.001	[0.579; 0.594]	0.751	0.004	<.001	[0.744; 0.758]
Partner's self-rated communal behavior	0.039	0.004	<.001	[0.031; 0.047]	0.052	0.004	<.001	[0.045; 0.059]
pnCommunion × Perception of partner's communal behavior	0.012	0.002	<.001	[0.008; 0.016]	0.001	0.002	.429	[-0.002; 0.005]
pnCommunion × Partner's self-rated communal behavior	-0.004	0.002	.044	[-0.009; <0.001]	-0.001	0.002	.716	[-0.004; 0.003]

Note. N = up to 40,853 surveys (global communal behavior) and up to 40,777 surveys (specific communal behaviors). Estimate = unstandardized regression coefficients. CI = 95% confidence intervals. Not displayed: effects of covariates weekend and time.

Table S5

Results of Multilevel Response Surface Analyses for the Prediction of Communal Need Satisfaction by Actors' and Partners' Self-Rated Communal Behavior

	Global communal behavior				Specific communal behaviors			
Effects	Estimate	*SE*	*p*	CI	Estimate	*SE*	*p*	CI
Effects								
Male intercept	4.358	0.084	<.001	[4.194; 4.522]	4.359	0.084	<.001	[4.195; 4.523]
Female intercept	4.274	0.080	<.001	[4.117; 4.430]	4.267	0.080	<.001	[4.110; 4.425]
Communal behavior (b_1)	0.312	0.007	<.001	[0.299; 0.325]	0.386	0.010	<.001	[0.367; 0.404]
Partner's communal behavior (b_2)	0.125	0.007	<.001	[0.112; 0.138]	0.193	0.010	<.001	[0.174; 0.212]
Communal behavior^2 (b_3)	<0.001	0.002	.961	[-0.005; 0.005]	-0.034	0.006	<.001	[-0.047; -0.022]
Communal behavior × Partner's communal behavior (b_4)	0.013	0.003	<.001	[0.007; 0.019]	-0.014	0.008	.083	[-0.031; 0.002]
Partner's communal behavior^2 (b_5)	-0.006	0.002	.013	[-0.011; -0.001]	-0.006	0.006	.361	[-0.018; 0.007]
RSA parameters								
a_1	0.437	0.009	<.001	[0.420; 0.454]	0.579	0.012	<.001	[0.556; 0.602]
a_2	0.007	0.004	.088	[-0.001; 0.014]	-0.054	0.010	<.001	[-0.074; -0.035]
a_3	0.187	0.010	<.001	[0.166; 0.207]	0.193	0.015	<.001	[0.163; 0.222]
a_4	-0.019	0.005	<.001	[-0.029; -0.008]	-0.026	0.014	.064	[-0.053; 0.001]

Note. N = 18,271 surveys (global communal behavior) and 18,260 surveys (specific communal behaviors). Estimate = unstandardized regression coefficients. CI = 95% confidence intervals. Not displayed: effects of covariates weekend and time.

Table S6

Results of Multilevel Response Surface Analyses for the Prediction of Communal Need Satisfaction by Communal Behavior, Perceptions of Partner's Communal Behavior, and Partner's Self-Rated Communal Behavior

	Global communal behavior				Specific communal behaviors			
Effects	Estimate	*SE*	*p*	CI	Estimate	*SE*	*p*	CI
Effects								
Male intercept	4.356	0.084	<.001	[4.191; 4.521]	4.420	0.083	<.001	[4.257; 4.584]
Female intercept	4.270	0.080	<.001	[4.113; 4.428]	4.326	0.080	<.001	[4.169; 4.483]
Communal behavior	0.167	0.009	<.001	[0.150; 0.184]	0.179	0.014	<.001	[0.152; 0.206]
Perception of Partner's communal behavior	0.216	0.009	<.001	[0.200; 0.233]	0.305	0.014	<.001	[0.277; 0.333]
Communal behavior^2	-0.011	0.003	.001	[-0.017; -0.005]	-0.072	0.010	<.001	[-0.092; -0.052]
Communal behavior × Perception of Partner's communal behavior	0.029	0.003	<.001	[0.023; 0.036]	0.146	0.016	<.001	[0.114; 0.178]
Perception of Partner's communal behavior^2	-0.014	0.002	<.001	[-0.019; -0.009]	-0.117	0.011	<.001	[-0.138; -0.095]
Partner's self-rated communal behavior	0.097	0.006	<.001	[0.084; 0.109]	0.152	0.010	<.001	[0.134; 0.171]
Partner's self-rated communal behavior^2	0.005	0.002	.065	[<0.001; 0.009]	-0.007	0.007	.324	[-0.020; 0.007]
Communal behavior × Partner's self-rated communal behavior	0.003	0.004	.405	[-0.004; 0.010]	-0.006	0.010	.503	[-0.025; 0.012]
RSA parameters (actual similarity)								
a_1	0.264	0.010	<.001	[0.243; 0.284]	0.332	0.017	<.001	[0.299; 0.364]
a_2	-0.003	0.005	.507	[-0.012; 0.006]	-0.085	0.014	<.001	[-0.112; -0.058]
a_3	0.071	0.011	<.001	[0.049; 0.092]	0.027	0.017	.113	[-0.006; 0.060]
a_4	-0.009	0.006	.128	[-0.021; 0.003]	-0.072	0.017	<.001	[-0.106; -0.038]
RSA parameters (perceived similarity)								
a_1	0.384	0.007	<.001	[0.370; 0.397]	0.484	0.010	<.001	[0.464; 0.504]
a_2	0.005	0.003	.079	[-0.001; 0.010]	-0.042	0.008	<.001	[-0.058; -0.026]
a_3	-0.049	0.016	.002	[-0.080; -0.019]	-0.126	0.026	<.001	[-0.177; -0.075]
a_4	-0.054	0.006	<.001	[-0.066; -0.041]	-0.335	0.032	<.001	[-0.397; -0.272]

Note. N = 18,271 surveys (global communal behavior) and 18,260 surveys (specific communal behaviors). Estimate = unstandardized regression coefficients. CI = 95% confidence intervals. Not displayed: effects of covariates weekend and time.

Table S7

Results of Multilevel Response Surface Analyses for the Prediction of Relationship Satisfaction by Actors' and Partners' Self-Rated Communal Behavior

	Global communal behavior				Specific communal behaviors			
Effects	Estimate	*SE*	*p*	CI	Estimate	*SE*	*p*	CI
Effects								
Male intercept	8.018	0.068	<.001	[7.886; 8.151]	7.986	0.070	<.001	[7.850; 8.123]
Female intercept	8.010	0.067	<.001	[7.878; 8.142]	7.967	0.069	<.001	[7.831; 8.103]
Communal behavior (b_1)	0.327	0.004	<.001	[0.319; 0.335]	0.346	0.006	<.001	[0.333; 0.358]
Partner's communal behavior (b_2)	0.135	0.004	<.001	[0.127; 0.143]	0.171	0.006	<.001	[0.159; 0.184]
Communal behavior^2 (b_3)	-0.030	0.002	<.001	[-0.033; -0.027]	-0.081	0.004	<.001	[-0.089; -0.072]
Communal behavior × Partner's communal behavior (b_4)	-0.009	0.002	<.001	[-0.013; -0.005]	0.003	0.006	.641	[-0.009; 0.014]
Partner's communal behavior^2 (b_5)	-0.021	0.002	<.001	[-0.024; -0.018]	-0.040	0.004	<.001	[-0.049; -0.032]
RSA parameters								
a_1	0.462	0.005	<.001	[0.452; 0.473]	0.517	0.008	<.001	[0.502; 0.532]
a_2	-0.059	0.002	<.001	[-0.064; -0.054]	-0.118	0.007	<.001	[-0.132; -0.105]
a_3	0.192	0.006	<.001	[0.180; 0.205]	0.175	0.010	<.001	[0.154; 0.195]
a_4	-0.042	0.003	<.001	[-0.048; -0.035]	-0.124	0.010	<.001	[-0.143; -0.105]

Note. N = 40,928 surveys (global communal behavior) and 40,866 surveys (specific communal behaviors). Estimate = unstandardized regression coefficients. CI = 95% confidence intervals. Not displayed: effects of covariates weekend and time.

Table S8

Results of Multilevel Response Surface Analyses for the Prediction of Relationship Satisfaction by Communal Behavior, Perceptions of Partner's Communal Behavior, and Partner's Self-Rated Communal Behavior

	Global communal behavior				Specific communal behaviors			
Effects	Estimate	*SE*	*p*	CI	Estimate	*SE*	*p*	CI
Effects								
Male intercept	8.054	0.068	<.001	[7.921; 8.186]	8.068	0.069	<.001	[7.932; 8.204]
Female intercept	8.054	0.066	<.001	[7.924; 8.183]	8.049	0.069	<.001	[7.914; 8.184]
Communal behavior	0.163	0.005	<.001	[0.153; 0.173]	0.101	0.009	<.001	[0.083; 0.119]
Perception of Partner's communal behavior	0.244	0.005	<.001	[0.234; 0.254]	0.356	0.009	<.001	[0.337; 0.374]
Communal behavior^2	-0.036	0.002	<.001	[-0.040; -0.033]	-0.109	0.007	<.001	[-0.123; -0.096]
Communal behavior × Perception of Partner's communal behavior	0.044	0.002	<.001	[0.040; 0.048]	0.188	0.011	<.001	[0.166; 0.210]
Perception of Partner's communal behavior^2	-0.048	0.001	<.001	[-0.051; -0.046]	-0.196	0.008	<.001	[-0.210; -0.181]
Partner's self-rated communal behavior	0.094	0.004	<.001	[0.086; 0.101]	0.125	0.006	<.001	[0.113; 0.138]
Partner's self-rated communal behavior^2	-0.007	0.001	<.001	[-0.010; -0.004]	-0.041	0.005	<.001	[-0.050; -0.031]
Communal behavior × Partner's self-rated communal behavior	-0.004	0.002	.096	[-0.008; 0.001]	0.028	0.007	<.001	[0.015; 0.041]
RSA parameters (actual similarity)								
a_1	0.257	0.006	<.001	[0.244; 0.269]	0.226	0.011	<.001	[0.205; 0.248]
a_2	-0.047	0.003	<.001	[-0.052; -0.042]	-0.122	0.009	<.001	[-0.140; -0.104]
a_3	0.069	0.007	<.001	[0.056; 0.082]	-0.025	0.011	.032	[-0.047; -0.002]
a_4	-0.040	0.004	<.001	[-0.047; -0.033]	-0.178	0.012	<.001	[-0.202; -0.154]
RSA parameters (perceived similarity)								
a_1	0.407	0.004	<.001	[0.399; 0.415]	0.456	0.007	<.001	[0.443; 0.470]
a_2	-0.040	0.002	<.001	[-0.044; -0.037]	-0.117	0.006	<.001	[-0.128; -0.106]
a_3	-0.081	0.009	<.001	[-0.099; -0.063]	-0.255	0.017	<.001	[-0.289; -0.221]
a_4	-0.129	0.004	<.001	[-0.136; -0.122]	-0.493	0.022	<.001	[-0.536; -0.450]

Note. N = 40,923 surveys (global communal behavior) and 40,847 surveys (specific communal behaviors). Estimate = unstandardized regression coefficients. CI = 95% confidence intervals. Not displayed: effects of covariates weekend and time.

Figure S1

Distribution of Discrepancies between Participants' Specific Communal Behaviors and Perceptions of their Partners' Specific Communal Behaviors

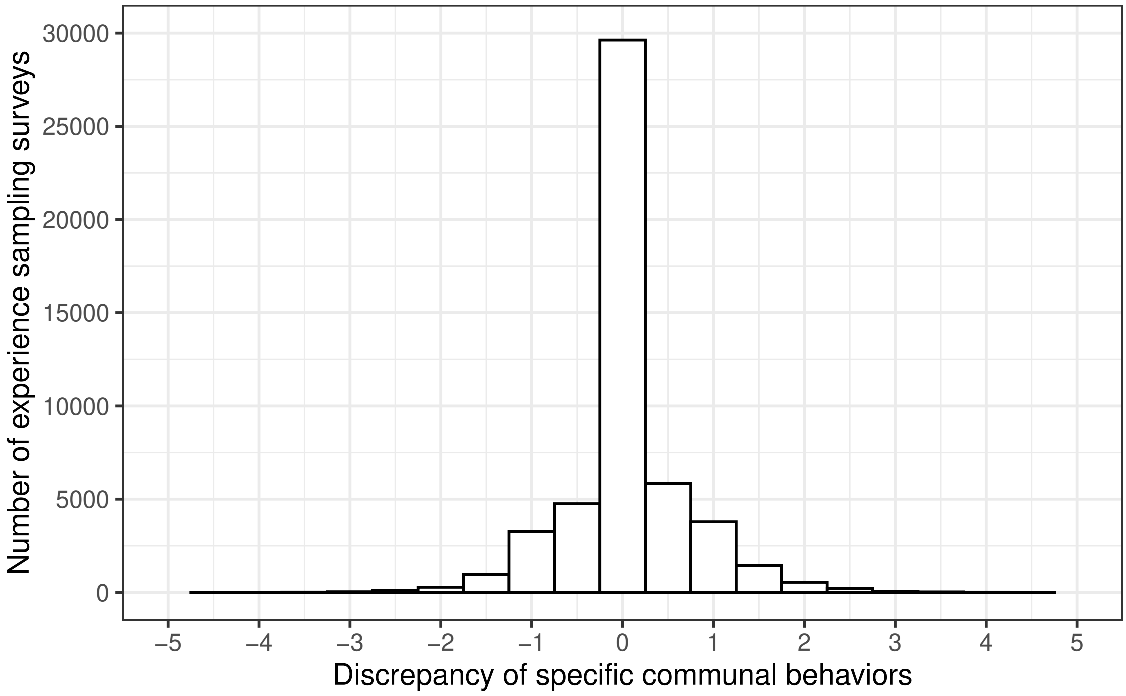

Note. Discrepancy values were computed by subtracting participants' specific communal behaviors from their perceptions of their partners' specific communal behaviors survey-wise. Figure available at https://osf.io/2fz5w/?view_only=8a0dc4c017e241a1987c12ca2f31fb13 under a CC-BY4.0 license.

Preceding Experience Sampling Study

The current study was preceded by an experience sampling study administered in November and December 2016 (Zygar et al., 2018b). Information about this preceding study (sample and procedures, measures) is provided in the following section. The data of this preceding study were not included in the current analyses because the preceding study employed slightly different measures to assess participants' momentary behavior (there was only a list eight specific communal behaviors participants could select from) and relationship satisfaction (assessed with only two items using a 7-point response scale). In addition, the preceding study was comparably shorter with only two weeks of experience sampling. However, to be fully transparent and to further substantiate the current findings, we re-ran our main analyses based on data from the preceding study.

With respect to the prediction global communal behavior (Hypothesis 1), the focal cross-level interactions between actors' communal motives and their perceptions of their partners' communal behavior no longer reached statistical significance (see Table S10). However, at least with regard to the analyses using actors' explicit desires for closeness as moderators, the confidence intervals of the interaction effects included the confidence intervals found in the main analyses, suggesting that discrepancy was due to the reduced statistical power in the preceding study.

The multilevel response surface analyses of communal need satisfaction (Hypothesis 2; see Table S11) and relationship satisfaction (Hypothesis 3; see Table S12) largely replicated our main findings. We found evidence for both positive main effects (a_1 response surface parameter) and positive similarity effects (negative a_4 parameter) of actors' communal behavior and perceptions of their partners' communal behavior on actors' communal need satisfaction and relationship satisfaction.

Sample and procedure

German couples were recruited in 2016 via local and online advertising. The study consisted of two parts. First, each member of a couple completed an online questionnaire set up via the formr survey framework (Arslan et al., 2019), in which their communal motive dispositions were assessed. Second, participants were asked to

download an experience sampling app (only usable on Android operating systems) on their smartphones. For the following two weeks, participants were asked to report on their momentary behavior and experiences via this app five times per day. Each day, data collection took place over a fixed period of 13.5 hours, which couples could schedule beforehand. The five daily survey invitations were sent out approximately equally distributed across this period, but the exact timing varied within a certain range to prevent expectancy effects. Both members of a couple received the survey invitations simultaneously but were asked to complete them on their own and not discuss any answers. Each survey was accessible for a time window of 45 minutes (median completion time: 3.28 minutes). After study completion, student participants could receive course credit and personalized feedback about their results. In addition, participants could register for a voucher lottery if they completed at least 80% of all daily surveys.

All participants who completed at least two thirds of the total 70 surveys were admissible to data analyses (see preregistered data exclusion criteria at https://osf.io/b8pu6/). Two participants were excluded because they were not in a couple relationship. The final sample consisted of 130 participants (51.5% female) nested in 68 couples. On average, participants were 22.75 years old (SD = 4.18; range: 18 to 40 years) and in the relationship with their current partners for 2.37 years (SD = 1.99; range: 3 weeks to 8.04 years). Only one participant had children, and the majority (79%) were university students. Overall, the response rate in the experience sampling was 85%, which resulted in data from up to 7,742 daily surveys.

Measures

Participants' communal motives (pnCommunion, explicit desire for closeness), global communal behavior, perceptions of their partner's global communal behavior, and communal need satisfaction were assessed analogously to the main study. However, only 8 behaviors were included in the list of specific communal behaviors (*paying particularly low regard to the partner* was not included in the preceding study). Moreover, the preceding study assessed relationship satisfaction with two items on a

7-point scale, without the main study item "How are you feeling at the moment over in your relationship?". Descriptive statistics of the preceding study data are presented in Table S9.

Table S9

Descriptive Statistics (Preceding Study)

	$M(SD)$ / $M_{\text{Grand}}(SD)$		$SD_{\text{Grand}}(SD)$		Between-person variance (%)		Within-person and error variance (%)	
Variables	Men	Women	Men	Women	Men	Women	Men	Women
pnCommunion	5.37 (2.19)	5.64 (2.11)	-	-	-	-	-	-
Desire for closeness	6.03 (0.74)	6.33 (0.60)	-	-	-	-	-	-
Global communal behavior	7.39 (1.11)	7.56 (1.06)	1.54 (0.44)	1.67 (0.48)	31.16	28.38	68.84	71.62
Perception of partner's global communal behavior	7.37 (1.08)	7.67 (1.09)	1.70 (0.58)	1.67 (0.53)	27.5	27.12	72.5	72.88
Specific communal behaviors	1.07 (0.62)	1.08 (0.56)	0.92 (0.28)	0.91 (0.24)	28.76	24.76	71.24	75.24
Perception of partner's specific communal behaviors	0.98 (0.59)	1.13 (0.58)	0.85 (0.25)	0.92 (0.25)	28.5	27.57	71.5	72.43
Communal need satisfaction	4.14 (1.18)	3.96 (1.30)	1.11 (0.42)	1.17 (0.32)	49.37	53.99	50.63	46.01
Relationship satisfaction	5.71 (0.54)	5.82 (0.58)	0.69 (0.31)	0.70 (0.27)	34.23	37.44	65.77	62.56

Note. N = up to 7,545 surveys of 130 individuals from 68 couples. M_{Grand} = Mean of individual person-means; SD_{Grand} = mean of individual person-standard deviations. Between-person variances correspond to intraclass correlations calculated via two-intercept models with random intercepts. Within-person and error variances correspond to the subtraction of the respective intraclass correlation from 1.

Table S10

Results of Multilevel Analyses for the Prediction of Communal Behavior by Perceptions of Partner's Communal Behavior and Communal Motives (Preceding Study)

	Global communal behavior				Specific communal behaviors			
Effects	Estimate	*SE*	*p*	CI	Estimate	*SE*	*p*	CI
**Explicit desire for closeness**								
Male intercept	7.347	0.131	<.001	[7.090; 7.604]	1.031	0.079	<.001	[0.877; 1.185]
Female intercept	7.361	0.128	<.001	[7.111; 7.612]	1.043	0.071	<.001	[0.904; 1.182]
Desire for closeness	0.525	0.127	<.001	[0.275; 0.775]	0.037	0.069	.592	[-0.098; 0.171]
Perception of partner's communal behavior	0.561	0.024	<.001	[0.514; 0.608]	0.831	0.013	<.001	[0.805; 0.857]
Desire for closeness × perception of partner's communal behavior	0.035	0.021	.098	[-0.006; 0.076]	0.014	0.015	.365	[-0.016; 0.044]
**Implicit pnCommunion**								
Male intercept	7.216	0.136	<.001	[6.950; 7.481]	1.018	0.077	<.001	[0.867; 1.168]
Female intercept	7.424	0.134	<.001	[7.162; 7.687]	1.036	0.069	<.001	[0.901; 1.171]
pnCommunion	0.112	0.045	.014	[0.024; 0.201]	0.057	0.023	.015	[0.012; 0.102]
Perception of partner's communal behavior	0.560	0.024	<.001	[0.512; 0.608]	0.832	0.013	<.001	[0.807; 0.858]
pnCommunion × perception of partner's communal behavior	-0.010	0.008	.181	[-0.026; 0.005]	0.007	0.004	.123	[-0.002; 0.016]

Note. N = up to 5,440 surveys (global communal behavior) and up to 7,457 surveys (specific communal behaviors). Estimate = unstandardized regression coefficients. CI = 95% confidence intervals. Not displayed: effects of covariates weekend and time.

Table S11

Results of Multilevel Response Surface Analyses for the Prediction of Communal Need Satisfaction by Communal Behavior and Perceptions of the Partner's Communal Behavior (Preceding Study)

	Global communal behavior				Specific communal behaviors			
Effects	Estimate	*SE*	*p*	CI	Estimate	*SE*	*p*	CI
Effects								
Male intercept	4.050	0.140	<.001	[3.775; 4.324]	3.872	0.151	<.001	[3.576; 4.168]
Female intercept	3.906	0.152	<.001	[3.607; 4.204]	3.695	0.161	<.001	[3.380; 4.010]
Communal behavior (b_1)	0.178	0.014	<.001	[0.151; 0.205]	0.205	0.025	<.001	[0.156; 0.255]
Perception of partner's communal behavior (b_2)	0.202	0.013	<.001	[0.176; 0.228]	0.318	0.026	<.001	[0.268; 0.368]
Communal behavior^2 (b_3)	-0.009	0.004	.043	[-0.017; <0.001]	-0.056	0.021	.008	[-0.098; -0.014]
Communal behavior × perception of partner's communal behavior (b_4)	0.019	0.005	<.001	[0.010; 0.029]	0.118	0.034	<.001	[0.052; 0.184]
Perception of partner's communal behavior^2 (b_5)	-0.004	0.004	.229	[-0.011; 0.003]	-0.045	0.023	.051	[-0.090; <0.001]
RSA parameters								
a_1	0.380	0.012	<.001	[0.357; 0.403]	0.523	0.015	<.001	[0.493; 0.553]
a_2	0.006	0.004	.152	[-0.002; 0.015]	0.017	0.012	.161	[-0.007; 0.041]
a_3	-0.024	0.024	.325	[-0.072; 0.024]	-0.113	0.048	.020	[-0.208; -0.018]
a_4	-0.032	0.009	<.001	[-0.049; -0.015]	-0.219	0.068	.001	[-0.352; -0.087]

Note. N = 5,409 surveys (global communal behavior) and 7,445 surveys (specific communal behaviors). Estimate = unstandardized regression coefficients. CI = 95% confidence intervals. Not displayed: effects of covariates weekend and time.

Table S12

Results of Multilevel Response Surface Analyses for the Prediction of Relationship Satisfaction by Communal Behavior and Perceptions of the Partner's Communal Behavior (Preceding Study)

	Global communal behavior				Specific communal behaviors			
Effects	Estimate	*SE*	*p*	CI	Estimate	*SE*	*p*	CI
Effects								
Male intercept	5.754	0.070	<.001	[5.616; 5.891]	5.643	0.071	<.001	[5.504; 5.781]
Female intercept	5.890	0.073	<.001	[5.748; 6.033]	5.768	0.073	<.001	[5.625; 5.912]
Communal behavior (b_1)	0.088	0.008	<.001	[0.072; 0.104]	0.038	0.017	.024	[0.005; 0.070]
Perception of partner's communal behavior (b_2)	0.135	0.008	<.001	[0.119; 0.150]	0.181	0.017	<.001	[0.148; 0.214]
Communal behavior^2 (b_3)	-0.022	0.003	<.001	[-0.027; -0.017]	-0.090	0.014	<.001	[-0.117; -0.062]
Communal behavior × perception of partner's communal behavior (b_4)	0.019	0.003	<.001	[0.013; 0.025]	0.172	0.022	<.001	[0.129; 0.216]
Perception of partner's communal behavior^2 (b_5)	-0.027	0.002	<.001	[-0.032; -0.023]	-0.100	0.015	<.001	[-0.130; -0.070]
RSA parameters								
a_1	0.222	0.007	<.001	[0.209; 0.236]	0.219	0.010	<.001	[0.199; 0.238]
a_2	-0.031	0.003	<.001	[-0.036; -0.026]	-0.017	0.008	.035	[-0.033; -0.001]
a_3	-0.047	0.015	.001	[-0.075; -0.018]	-0.144	0.032	<.001	[-0.206; -0.081]
a_4	-0.069	0.005	<.001	[-0.079; -0.058]	-0.362	0.044	<.001	[-0.449; -0.275]

Note. N = 5,440 surveys (global communal behavior) and 7,457 (specific communal behaviors). Estimate = unstandardized regression coefficients. CI = 95% confidence intervals. Not displayed: effects of covariates weekend and time.
